# Tribological Response of Basalt/Carbon Hybrid Laminated Composites After Water-Immersion Aging: Influence of Stacking Sequence Under Reciprocating Wear

**DOI:** 10.3390/polym18010057

**Published:** 2025-12-25

**Authors:** Sinan Fidan, Satılmış Ürgün, Mehmet İskender Özsoy, Mustafa Özgür Bora, Togayhan Kutluk, Erman Güleç

**Affiliations:** 1 Department of Airframe and Powerplant Maintenance, Faculty of Aeronautics and Astronautics, Kocaeli University, Kocaeli 41001, Türkiye; sfidan@kocaeli.edu.tr (S.F.); ozgur.bora@kocaeli.edu.tr (M.Ö.B.); 2 Department of Aviation Electrics and Electronics, Faculty of Aeronautics and Astronautics, Kocaeli University, Kocaeli 41001, Türkiye; urgun@kocaeli.edu.tr; 3 Department of Mechanical Engineering, Faculty of Engineering, Sakarya University, Sakarya 54050, Türkiye; 4 Department of Chemical Engineering, Faculty of Engineering, Kocaeli University, Kocaeli 41001, Türkiye; togayhan.kutluk@kocaeli.edu.tr; 5 Otokar Automotive Defense Industry Corp., Sakarya 54580, Turkey; egulec@otokar.com.tr

**Keywords:** hybrid basalt–carbon fiber laminates, pressurized water-immersion aging, reciprocating wear behavior, stacking sequence effects, tribology

## Abstract

This study investigates the tribological response of basalt, carbon, and basalt–carbon hybrid laminates subjected to pressurized water-immersion aging and reciprocating sliding, with emphasis on the role of stacking sequence. Composite plates with B_8_, C_8_, B_2_C_4_B_2_, and C_2_B_4_C_2_ architectures were aged in deionized water at 10 bar for up to 30 days, then tested against a 100Cr6 steel ball at 30 N, 50 m track and 1 or 2 Hz. Water uptake ranged from approximately 4.3% for B8 to 1.2–2.7% for carbon-rich and hybrid laminates, and induced a depression and broadening of the epoxy glass-transition region that was most severe in basalt-skinned systems. At 1 Hz and 30-day aging, B8 exhibited the most severe damage, with wear-scar widths and depths approaching 3.0 mm and 0.50 mm, whereas C8 retained narrow shallow scars below 0.8 mm and 0.02 mm and COF values below 0.20. Increasing frequency to 2 Hz mitigated wear, reducing B8 depth to approximately 0.30 mm under similar conditions. Factorial analysis attributed more than 70% of the variance in wear width to laminate architecture. The combined pressurized immersion, multi-frequency reciprocating wear and DSC, profilometry, and SEM methodology provides an original framework to link hygrothermal plasticization to architecture-dependent tribological durability in hybrid basalt–carbon laminates.

## 1. Introduction

Hybrid composites are designed to combine two or more varying materials or mixtures of materials and fillers into matrices. This careful design provides optimal suitability for certain applications. This deliberate design, where varied components are inserted to enhance the global properties of the new composite, provides better mechanical, thermal, and tribological properties, including outstanding frictional resistance and wear resistance capabilities [1,2,3,4,5]. Hybrid composites are ideal in erosive environments where materials experience erosive wear or shedding as a result of impaction by eroding materials or particles present in environments, for example, dust, sand, or slurries [6,7]. It has been observed and analyzed in existing research studies that hybrid modification between other variation types or other kinds of varied materials or other varied compositions within these composite materials offers minimized disadvantages, so they present better and larger benefits or advantages altogether. The reduced frictional resistances between material-processed layers or layers with varied materials utilizing FRP present great attraction for research or modifications to bearing, gear, or brake parts or products for frictional uses. FRP demonstrates bright features for reduced mass uses but has drawbacks in terms of higher or sometimes relatively expensive costs, which act as limited factors for its or their general adoption or acceptance for common or general uses and applications into or within industries or society in general [8,9].

Basalt fibers can be classified as natural materials that are environment-friendly, possessing remarkable characteristics such as strength, resistance to chemicals, and resistance to high temperatures and ultraviolet radiation [10,11]. As a consequence, they are increasingly regarded as alternatives to glass fibers [12,13]. Moreover, recent studies also investigated the hybridization of basalt and carbon fibers to produce advanced composite materials [14]. Sahin and De Baets [15] studied dry wear resistance for hybrid composites with various stacking sequences of basalt and carbon fabrics with compositions HB1, HB3, and HC3 using a pin-on-plate setup. Wear resistance decreased substantially with increasing sliding speed, which was more severe than for other factors. The HC3 composite with three layers of exterior carbon layers presented better wear resistance due to its superior mechanical properties. Scanning electron microscopy observations on worn surfaces provided evidence for fracture in HB1, predominantly matrix wear for HB3 at higher speeds, and craters together with deformed mixed layers on top for HC3. Darshan et al. [16] studied three body abrasions on silk/basalt-epoxy matrix compositions reinforced with Halloysite Nanotubes (HNTs), tackling “sustainable material needs.” Employing ASTM G65-04 [17] Rubber Wheel Testing and using Taguchi L27 design, they depicted how various parameters, including filler content, load, abrasing distance, and size of sand particles, affected wear rates to provide needed data. Results demonstrated dramatic increases in wear resistance via HNT addition. Analysis of variance revealed the important variables to be load, abrasing distance, filler content, and sand size, in sequence. Kim and others [18] studied how adding 1 wt.% CNT treated with “sulfuric acid” to basalt-epoxy matrices influenced wear-resistant properties and dynamic-thermo-modal or dynamic mechanical-thermo analytic behavior to explore its feasibility for industrial applications. Addition of CNTs substantially raised performances by reducing friction factors to 0.3–0.4 and reducing wear volume losses by about 68% for pure basalt-epoxy matrix compositions. Moreover, an additional further raised glass transition temperature for dynamic properties is assumed to better transfer loads evenly between epoxy matrices and reinforcement layers provided by CNTs, stabilizing the matrix structure. Chairman and Babu [19] probed into wear features of glass and basalt-epoxy matrix compositions using various woven fabrics manufactured via “hand laying.” Characteristics of mechanical properties and abrasive wear investigations using a pin-on-disc apparatus with 400-girt SiC sheets at room temperature also took place. It was shown that interfacial bonding between basalt and matrix has a beneficial effect on abrasive wear, where the addition of basalt fibers increases abrasive wear resistance.

Industries are also interested in addressing tribological problems in moving parts to achieve cost savings, improve efficiencies, and ensure environmental compliance. Water absorption is also important to take into consideration in material selection, as it can contribute to degraded mechanical properties, plasticization, or swelling that can alter friction and wear properties between polymers and other materials, thereby optimizing polymer part life [20,21]. Ramie and carbon hybrids with different sequences had their hygro-thermal aging and flexure evaluated. After 90 days of immersion in water, moisture content varied between 2.36% and 2.72%, which was authenticated by finite element simulation. Flexure strength reduced by 25.2–36.9%, with interfacial transitions from brittle to ductile failure. The ([C/C/R/R]s) arrangement provided better flexure modulus properties than others [22]. Özsoy et al. [23] analyzed the bi-directional reciprocating slide currents for glass & Kevlar hybrid materials immersed in pressurized water. Samples absorbed up to 8.54% in 21 days, adhering to Fickian law. Friction coefficient reached its minimum on day 14, showing a reduction of 20–40% compared to that to be followed later for unaged specimens. Wear volume raised with immersion time, as shown by profilometry analyses, where widths varied between 1596–2101 μm and depths between 128–185 μm. Scanning electron microprobe analyses disclosed transitions to matrix plasticization and then to fiber debonding.

An assessment on nanoscale interface debonding and its energy release rates in polymer matrix composites after two years of aging under hygrothermal environments at 60 °C and 90% relative humidity was conducted by Fard et al. [24]. The nanoscale interface debonding presented a mixed failure mode between cohesive and adhesive failures. Moreover, degradation in interphases with a reduction in macroscale fracture initiation threshold, but with increased mode I fracture threshold for the aged specimen, was observed in the initial year. Debonding within interfaces and nanoscale cracks promoted cracks within intralamina, including fiber-bridging, with relatively higher energy for crack growth. Fabrication and evaluation for glass/epoxy, carbon/epoxy, and hybrid laminated composites for aviation applications were described by Guermazi et al. [25]. After aging, substantial moisture absorption was observed for all composites, but glass/epoxy absorbed higher percentages than the other two types. Moreover, according to thermogravimetric analysis, hybrid composite possessed higher thermal stability than others. Mechanically and for wear resistance, carbon-epoxy had highest values, while for hybrid laminate, they possessed medium ranks. Hygrothermal aging reduced strength for all materials, but for carbon-epoxy, they possessed minimum susceptibility to aging damage after aging for 90 days at 90 °C. Experimental data for glass-epoxy (G-E), carbon-epoxy (C-E), and hybrid laminated composites to design repairing aircraft using composite materials under hygrothermal subjects was presented by authors Guermazi et al. in [26]. Experimental observations depicted that each composite followed the Fickian profile for moisture absorption, though glass-epoxy possessed the highest ability to absorb moisture with its hydrophilic nature. Carbon-epoxy possessed highest resistance to wear, though glass-epoxy possessed highest sensitivity to aging degradation.

Sukur et al. [27] investigate how silica (SiO_2_), halloysite (HNT), or montmorillonite (NC) nanoparticles affect damage tolerance properties in epoxy composites reinforced with basalt fibers exposed to hygrothermal aging. Adding 2 wt.% nanoparticles to enhance mechanical properties improved tensile strength for SiO_2_ by 44% and flexural strength for HNT-modified samples by 30%. Hygrothermal aging slightly raised impact resistance, and HNT-modified samples demonstrated maximum improvement by 8%. DMA analysis and FTIR studies reported increases in crosslink density, elastic modulus, and glass transition temperature resulting from physical aging. Hynt-modified samples also featured 17.8% increases in compression after impact strength. Li et al. [28] discuss how cerium chloride (CeCl_3_) surface-treated basalt fibers improve their own and epoxy composites’ impact on environmental factors, i.e., hygrothermal stability for modified basalt/epoxy composites (BF/ERCs). Their modified basalt/epoxy matrices featured analyses for water sorption properties, tensile strength, flexure strength, and interlaminar shear strength for modified basalt matrices prior to aging. Their work reveals modification using 0.5 wt.% Ce chloride solution minimized absorptive properties and ensured sustained mechanical properties to its fullest extent, therefore improving its durability against environmental aging. Bonsu et al. [29] analyze glass, basalt, and glass-basalt t hybrid matrices’ failures under seawater aging environments at either 30 °C or 70 °C for 258 days. GB3 ((B_2_G_2_)S) hybrids containing outer layers composed of basalt instead demonstrated highest retention in tension and flexure strength by 100% and 86.6% after aging, while ‘GB4 S’ hybrids, possessing highest ‘lateral’ impact resistance, possessed cracks within hybrids that minimized failures to generate the strongest hybrids instead. Scanning electron microscopy also analyzed basalt matrices’ failure after immersion in seawater aging, and impeded failures and cracks within their glass hybrids instead. Kumar & Bhowmik [30] investigate kenaf/pineapple laminate bio-epoxy bio composites as environmentally friendly alternatives for structural usage. The hybrid bio composites exhibit improved interfacial bonding, compactness, rougher surface morphology, and better thermo-mechanical properties with improved thermal stability. However, severe weathering causes decreased surface hardness and elevated hydrophilicity and roughness. Friction and wear studies under various normal loads and humid environments reveal substantial volume loss, elevated frictional force, and raised rates during weathering and humid environments. The analysis of worn-out surfaces also detects interfacial delamination and formation of cavities. Yorseng et al. [31] designed bio-based hybrid bio composites using kenaf and sisal fabrics with bio-epoxy matrix using compression molding. The hybrid bio composites are also tested prior to and post-weathering treatment. The studies reveal that hybrid bio composites possess moderate but relatively stable properties comparable to those of neat bio-epoxy bio composites prior to and post-weathering treatment. Despite its hydrophilic nature, bio-based hybrid bio composites possess promising usage for semi-structural materials as sustainable alternatives to synthetic materials.

Plasticization due to the absorption of water present in epoxy-based composites is proven to be a predominantly important mechanism of hygrothermal effects, and it is found to be accurately revealed due to the decrease in the glass transition temperature (Tg) [32,33]. This quantitative expression of Tg and moisture absorption has been quantitatively explained by Starkova et al. [34] on epoxy/MWCNT composites, showing a nominal decrease of about 20 °C with 2.5% of moisture, which is extremely sensitive to about 8 °C/per 1% of moisture. Similarly, another important study has been conducted by Ghabezi and Harrison [35] on carbon-epoxy and glass-epoxy laminates, which were subjected to simulated seawater at 60 °C for 180 days. An important fall in Tg value has been noted, which further verifies the plasticization of the epoxy due to the presence of moisture. Contrary to it, hot water aging at 50–70 °C has been investigated by Russell et al. [36] on cotton/E-glass hybrid composites. After 180 days, DSC analysis verified Tg, as well as cross-link density, and it has been found to increase due to hot water aging, which further highlights the plasticizing and post-curing reaction of the interpenetrating polymer matrix. Zulueta et al. [37] studied the influence of hot water aging on carbon fiber reinforced C-SMC materials. After hot water aging at 80 °C and 65 °C RH, a nominal value of 1.13% has been achieved, following Fick’s laws. DMA analysis revealed a nominal fall of 27 °C due to plasticization. Tensile, bending, and interlaminar shear strength tests revealed insignificant changes, which further verified hot water aging. Another important study has been conducted by Lu et al. [38] on T700 carbon fibers and T700 carbon-epoxy composites, which were subjected to long hot water aging up to 700 days with seawater. Nominal value of 0.35% attained due to moisture, following Fick’s laws. Experimental evidence and simulations revealed that the value of the longitudinal elastic modulus, which is predominantly dependent on fiber, is barely affected, at a level of 10^−5^%, compared to 10^−3^% of the transverse and shear moduli, which are largely dependent on the epoxy matrix. Moreover, the increase in exposure temperatures, closer to the Tg value of the matrix, resulted in an abrupt decline of the matrix-dependent mechanical properties by up to 100%, thereby emphasizing the importance of exposure temperatures during marine and environmental service conditions.

Given the existence of existing literature mainly exploring the aging behaviors and frictional properties at atmospheric pressure or exploring new pairs of reinforcements, there exists a remarkable research gap about the reciprocating-sliding wear behavior of basalt-carbon hybrid laminates immersed in pressurized water. To address this research gap, the present study systematically investigates the combined influence of specific stacking sequences ([B_8_], [C_8_], [B_2_C_4_B_2_], and [C_2_B_4_C_2_]) and pressurized hygrothermal aging on the tribological behavior of woven epoxy composites reinforced with basalt and carbon fibers. Unlike conventional studies that predominantly rely on standardized tribological methods such as pin-on-disc or abrasive wear tests, this research adopts a multi-faceted experimental approach to achieve a more comprehensive evaluation of wear mechanisms and frictional behavior under complex environmental conditions. Furthermore, to quantitatively assess matrix-level plasticization induced by moisture absorption, DSC analyses were systematically performed to monitor variations in the glass transition temperature (T_g_). This integrated thermo-mechanical and tribological characterization framework enables a direct correlation between hygrothermal aging–induced matrix softening and the resulting changes in tribological performance. Moreover, instead of restricting to existing simplistic research tropes that mainly focus on existing graphical outputs or other characterization studies relying on simplistic Curtains-cleaning-fracture-disintegration procedures, this research work investigates this topic from both qualitative and quantitative spectrums using advanced research characterization approaches focusing on 3D profilometry studies for wear mechanism characterization as well as Scanning Electron Microscopes for post-SEM studies on wear topography characterization.

## 2. Materials and Methods

### 2.1. Materials

**Figure 1:**
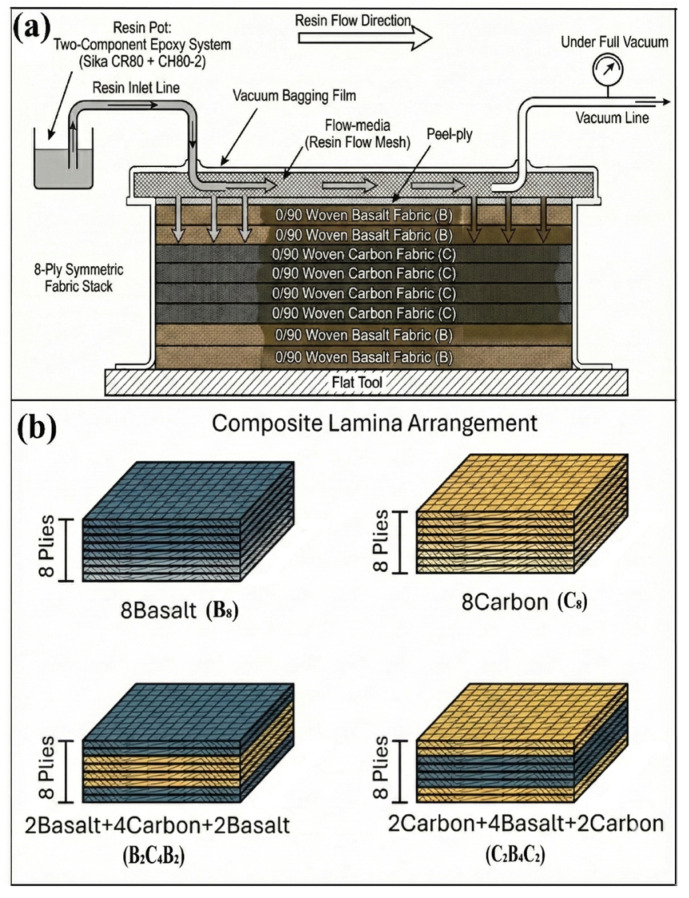
(**a**) Schematic illustration of the vacuum infusion process. (**b**) composite lamina stacking sequence arrangement.

Composite laminates were fabricated through vacuum infusion utilizing a two-component epoxy system, SikaBiresin brand CR80 type resin with CH80-2 type hardener, which has a 100:30 mixing ratio by weight; supplied by Tekno Endüstriyel Kimyasallar, İstanbul, Türkiye) as the matrix. The resin exhibits a density of 1.01 g mL^−1^, a tensile strength of 83 MPa, a tensile modulus of 2900 MPa, a Shore-D hardness of 84, and a glass-transition temperature of 93 °C according to supplier data sheets. Reinforcements consisted of 0/90 woven carbon and basalt fabrics, each with an areal density of 200 g m^−2^ (provided from Dost Chemical Corp., İstanbul, Türkiye). Supplier data reveal that the fibers’ properties, such as the carbon fiber exhibits a tensile strength of 3950 MPa, an elastic modulus of 238 GPa, a density of 1.76 g cm^−3^, a thermal conductivity of 17 W m^−1^ K^−1^, and a coefficient of thermal expansion (CTE) of −0.1 × 10^−6^ °C^−1^. Basalt fibers demonstrate a tensile strength of approximately 3100 MPa, an elastic modulus ranging from 88 to 92 GPa, a density between 2.60 and 2.63 g cm^−3^, thermal conductivity of 0.031 to 0.038 W m^−1^ K^−1^, and a coefficient of thermal expansion of 8 × 10^−6^ °C^−1^. Illustration of the composite molding process carried out by the vacuum infusion technique, as shown in Figure 1. Panels measuring 400 × 400 mm^2^ were infused on a flat tool under full vacuum, utilizing standard peel-ply and flow media consumables. Polivaks N PVA wax and Polivaks SIVI PVA were used on the tool surfaces, respectively, as the release agents, which were supplied by Poliya (İstanbul, Türkiye). During the vacuum infusion process, approximately 0.95 bar was applied, which corresponds to close to the atmospheric pressure. Four eight-ply, symmetric stacking architectures were produced as illustrated in Figure 1b: all-basalt (B_8_), all-carbon (C_8_), basalt-skinned hybrid (B_2_C_4_B_2_), and carbon-skinned hybrid (C_2_B_4_C_2_). Symmetry reduces extension–bending coupling, whereas the selection of outer skin material (basalt versus carbon) facilitates the comparison of skin-dominated responses. Following an initial curing period of 24 h at room temperature, the laminates were post-cured at 60 °C for 4 h. For each laminate configuration, three specimens were fabricated to ensure repeatability and reliability of the experimental results. Specimens with dimensions of 50 mm × 50 mm were cut using a water-jet machine for wear tests and microstructural characterization.

### 2.2. Procedure for Reciprocating Wear Tests

**Figure 2:**
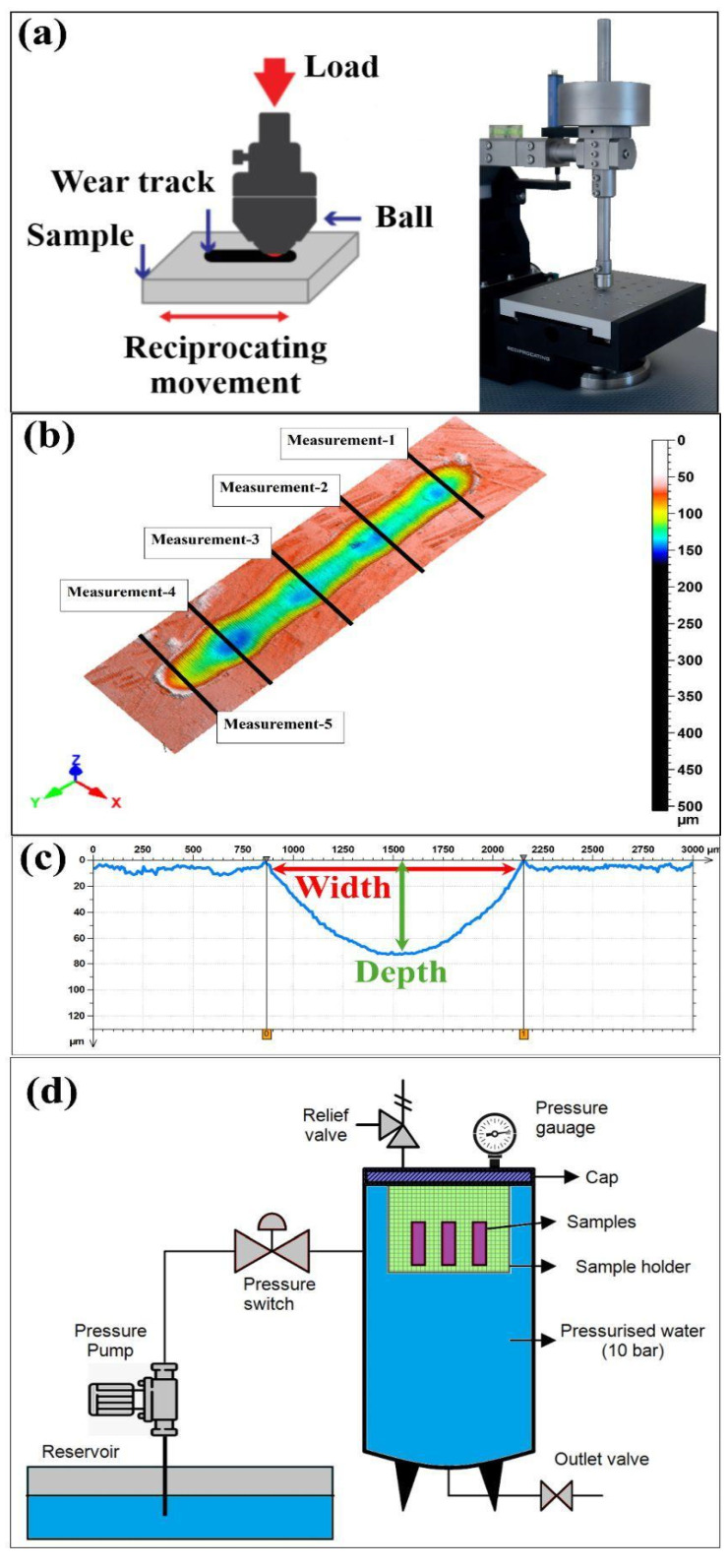
Experimental setup used in reciprocating wear tests and water-immersion aging: (**a**) re-ciprocating wear test setup, (**b**) wear track 3D measurements taken from reciprocating wear track, (**c**) wear track width and depth measurements taken from reciprocating wear track, (**d**) pressurized water aging test setup.

Using the UTS Tribolog^TM^ Multi-Function Tribometer (Istanbul, Türkiye) in reciprocating mode (Figure 2a), a stationary 6 mm 100Cr6 bearing steel ball (60–66 HRC; as-supplied surface roughness Ra < 0.05 µm) was pressed against the flat composite specimen under a constant normal load of 30 N, while the specimen oscillated linearly to generate an elongated wear scar on the laminate surface. Reciprocating motion was applied at test frequencies of 1 and 2 Hz, and the stroke length together with the number of cycles was selected such that 2 × strokes × cycles = 50 m, ensuring a prescribed total sliding distance of 50 m for each frequency and aging condition (Table 1). For each stacking sequence (B_8_, C_8_, B_2_C_4_B_2,_ and C_2_B_4_C_2_), unaged coupons (0-day) and specimens subjected to water-immersion aging for 10-day, 20-day, and 30-day were tested with a fresh track generated for every individual parameter set to avoid cross-interaction between wear scars. Prior to testing, all specimens were conditioned for 24 h at 23 ± 2 °C and 50 ± 5% relative humidity, and the tests were carried out under the same ambient conditions. The evolution of the friction coefficient as a function of sliding distance was recorded continuously by the tribometer data acquisition system, and the resulting wear track on each composite surface was subsequently examined for comparative assessment of the combined effects of stacking sequence, test frequency, and water-aging duration on the reciprocating wear response.

### 2.3. Water Immersion

**Figure 3:**
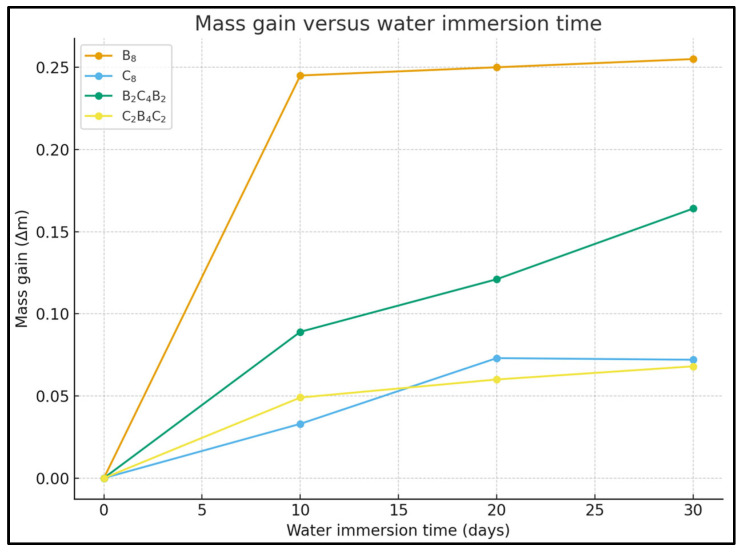
Mass increase (Δm) and corresponding water uptake percentage changes with time of composite samples during pressurized water immersion aging. Values measured over the 0–30 day period for B_8_, C_8_, B_2_C_4_B_2,_ and C_2_B_4_C_2_ laminates with different fiber architectures show the effect of the layer sequence on the water diffusion rate and saturation tendency.

The pressurized water immersion aging was carried out in a closed chamber, using deionized water held at 25 ± 1 °C and controlled at 10.0 ± 0.1 bar using a pump and pressure switch cycle (Figure 2d). The samples were supported on a PTFE holder and completely immersed in the water for periods of 0-day, 10-day, 20-day, and 30-day. After each aging duration, the samples were slowly depressurized. Prior to tribological testing, the surfaces were dried and then subjected to 24-h conditions of 23 ± 2 °C and 50 ± 5% RH. The choice of the applied pressure of 10 bar was intended to represent the conditions under working pressures, where the samples would work under high pressures, and hence increased water uptake would be achievable at 25 ± 1 °C, and prevent cavitation and temperature drift. The ageing times of 10-day, 20-day, and 30-day were chosen to represent the transient, intermediate, and saturation phases, respectively, in the water uptake curve. Figure 3 below shows the increase in masses and water uptake percentage. The B8 samples absorbed high water, up to 4.22%, on the 10th day, and the uptake revealed a high saturation rate, up to 4.28%, on the 30th day. The C8 sample showed lower water absorption, 0.57%, on the 10th day and approximately 1.25% on the 30th day, denoting the diffusion resistance characteristic and the barrier effect exerted by the carbon fibers.

The water absorption in the hybrid laminates was strongly dependent on the arrangement of the layers. When the basalt-carbon and basalt layers were arranged in the B_2_C_4_B_2_ manner, the water change was 1.48%, 1.95%, and 2.66% on the 10th, 20th, and 30th days, respectively, and in the C_2_B_4_C_2_ arrangement, the values were 0.84%, 1.08%, and 1.25%, respectively, on the 10th, 20th, and 30th days, respectively, demonstrating the effect of the arrangement on the ease of water flow. The water change was lowest in the hybrid laminates, where the outer layers were made up of carbon, and this was reflected in the stable performance in the pressurized water immersion test. Figure 3 shows the effect of the fibers and arrangement on the water change and the corresponding percentage increase in weight.

### 2.4. Surface Topography Characterization by Non-Contact Laser Profilometer

A non-contact laser profilometer (Nanovea PS-50) was utilized to measure the three-dimensional surface texture of the all-basalt (B_8_), all-carbon (C_8_), basalt-skinned hybrid (B_2_C_4_B_2_), and carbon-skinned hybrid (C_2_B_4_C_2_) composite specimens after reciprocating wear tests. This optical technique does not involve probe–surface interaction and allows for true visualization and extraction of the roughness parameters of the wear area. Raw height data were analyzed using DigitalSurf software (application-only, version 6.2.7487; Mountains Technology), and 3D areal texture parameters were calculated in accordance with ISO 25178 [39]. For each reciprocating wear scar, five individual measurements were acquired with the non-contact laser profilometer along the wear track, and the corresponding width and depth values were determined from the arithmetic mean of these repeated measurements. Representative three-dimensional surface maps and cross-sectional profiles used for the quantitative evaluation of wear scar width and depth are shown in Figure 2b and Figure 2c, respectively, illustrating the typical morphology of the reciprocating wear tracks on the different laminate configurations.

### 2.5. Scanning Electron Microscope (SEM) Examination

Surface damage occurring during reciprocating sliding tests was characterized using a JEOL JSM-6060LV scanning electron microscope (SEM), JEOL (JSM-6060LV, Tokyo, Japan). SEM examination of the wear tracks enabled detailed evaluation of the damage morphology and fiber-matrix interactions, while also allowing identification of the wear mechanisms governing the tribological behavior of the composite laminates.

### 2.6. Differential Scanning Calorimetry (DSC) Analysis

Differential Scanning Calorimetry (DSC) analyses were conducted to investigate the influence of water immersion aging on the glass transition temperature (Tg) of all-basalt (B_8_), all-carbon (C_8_), basalt-hybrid (B_2_C_4_B_2_), and carbon-hybrid (C_2_B_4_C_2_) composite laminates, both before and after aging. Following the prescribed aging periods, specimens were sectioned from the laminates, while additional reference samples were prepared from untested specimens aged for 0 and 30 days. Each specimen was carefully trimmed to eliminate surface debris and subsequently weighed to obtain a mass in the range of 5–10 mg before being hermetically sealed in standard aluminum DSC pans.

DSC measurements were performed under a nitrogen purge atmosphere within a temperature range selected to encompass the glass transition region of the epoxy matrix as well as any post-curing–related exothermic reactions. The analyses were carried out using a Mettler Toledo DSC 1 STAR System under a constant nitrogen flow rate of 20 mL min^−1^, with a heating rate of 10 °C min^−1^ over the temperature interval of 25–200 °C. All DSC tests were conducted in accordance with ASTM D3418-21 [40].

## 3. Results

### 3.1. COF Analysis of Reciprocating Wear Tests

**Figure 4:**
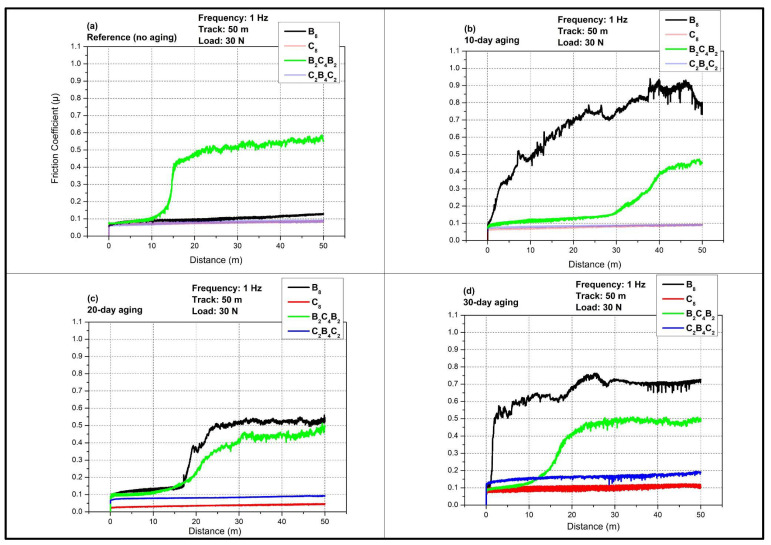
Effect of water aging time on COF values at 30 N load, 50 m track at 1Hz in reciprocating wear tests: (**a**) Reference (no aging); (**b**) 10-day aging; (**c**) 20-day aging; (**d**) 30-day aging.

Figure 4 presents the evolution of the coefficient of friction (COF, µ) with sliding distance for all stacking sequences tested at 1 Hz under a 30 N normal load and a total reciprocating track length of 50 m after different pressurized water-immersion durations. The curves display a short running-in stage followed by a quasi-steady-state regime whose level and stability are strongly affected by the extent of water aging and by whether the outer skins are basalt or carbon. Comparison of the four panels demonstrates that water immersion induces a pronounced increase and destabilization of friction for the all-basalt (B_8_) and basalt-skinned hybrid (B_2_C_4_B_2_) laminates, whereas the all-carbon (C_8_) and carbon-skinned hybrid (C_2_B_4_C_2_) systems remain comparatively stable up to intermediate aging times, consistent with their lower water uptake reported in Figure 3.

In the unaged reference state (Figure 4a), all laminates exhibit a low COF during the initial running-in period, with µ starting around 0.04–0.06 for the first few meters of sliding. The C_8_ and C_2_B_4_C_2_ laminates maintain a relatively flat friction response over the entire 50 m track, and the steady-state COF remains below about 0.10, indicating a predominantly mild adhesive or mixed adhesive–abrasive regime compatible with the high stiffness and smooth surface of carbon outer plies. The B_8_ laminate shows a slightly higher but still stable COF, rising gently toward values close to 0.10 with limited fluctuations, which can be related to the lower hardness and higher roughness of basalt fibers compared with carbon. The most distinctive behavior in the reference condition is observed for the B_2_C_4_B_2_ laminate. After a short low-friction running-in region similar to the other laminates, the green curve exhibits a sharp transition around 10–15 m to a higher plateau where µ reaches about 0.45–0.55 and remains nearly constant. This step-like increase suggests a breakthrough of the resin-rich top layer and the onset of more severe ploughing and microfracture of the basalt-rich surface, which dominates the steady-state friction in the unaged state for this stacking sequence. This abrupt transition is specific to the hybrid B_2_C_4_B_2_ architecture, where basalt outer plies are combined with a carbon core. During vacuum infusion, dissimilar fabric compaction and local permeability between basalt and carbon layers can promote a thin resin-rich, relatively smooth surface film on the basalt skins and local resin accumulation around near-surface tows. In the early sliding stage, this matrix-dominated contact yields a low COF comparable to carbon-skinned systems; once the weak resin-rich layer is removed, the contact transitions rapidly to a fiber-controlled regime, where protruding basalt tows become load-bearing and promote microfracture and three-body ploughing, producing the observed step-like COF increase. In contrast, the monolithic B_8_ laminate presents basalt fibers as the dominant near-surface phase from the beginning, so the matrix-to-fiber transition occurs more progressively and does not manifest as a discrete ‘breakthrough’ jump.

After 10 days of pressurized water immersion (Figure 4b), the frictional response of the laminates becomes strongly differentiated. Because B_2_C_4_B_2_ always presents basalt skins at the sliding interface, any moisture uptake preferentially plasticizes the near-surface epoxy and weakens basalt–epoxy interfacial adhesion, thus promoting earlier debris generation and a systematic COF increase with ageing time, even though the onset distance and severity remain lower than B_8_ due to the smaller absolute moisture uptake of B_2_C_4_B_2_. The B_8_ laminate shows the most detrimental response. Its COF increases rapidly after the running-in stage and exhibits a continuous rise with pronounced stick–slip fluctuations, reaching peak values close to 0.8 toward the end of the 50 m track. This behavior indicates extensive matrix plasticization and interfacial degradation due to the high-water uptake of the all-basalt system, which promotes a larger real contact area and facilitates microcracking and fiber/matrix debonding that enhance ploughing and third-body formation. The B_2_C_4_B_2_ laminate also shows a marked increase relative to the reference case, although less dramatic than B_8_. Its COF remains low over the initial portion of the track, then gradually climbs to about 0.4–0.45 after roughly 30–40 m, pointing to progressive damage accumulation and exposure of the inner plies as the test proceeds. In contrast, the C_8_ and C_2_B_4_C_2_ laminates preserve a low and stable COF close to their unaged levels, with only minor increases and limited noise. This confirms the role as a protective overlayer of carbon, acting effectively against the entry of water and reducing the softening effect on the matrix, keeping the surfaces in a smoother, stable condition in contact with the counter face after short-term aging.

At 20 days of water immersion (Figure 4c), the frictional response of the basalt-dominated laminates partially relaxes compared with the 10-day condition. For B_8_, the COF still displays a distinct transition from the initial low-friction regime to a higher plateau, yet the steady-state level stabilizes around 0.45–0.50 instead of approaching 0.8. The reduced amplitude of fluctuations suggests that the near-surface matrix has reached a more homogeneous plasticized state and that some of the weakly bonded or highly damaged material removed during prior sliding allows the system to enter a more stable, although still high, friction regime. The B_2_C_4_B_2_ laminate shows a similar stepped behavior, with the COF rising after about 15 m to a plateau slightly below that of B_8_, consistent with its intermediate water uptake. The carbon-containing laminates remain comparatively insensitive to this intermediate aging duration. Both C_8_ and C_2_B_4_C_2_ maintain low COF values with only a modest upward shift in the steady-state level and minimal noise, which indicates that the degree of matrix plasticization and interfacial weakening in these architectures remains limited even after 20 days, and the wear mechanism is still dominated by mild adhesive sliding with restricted micro ploughing.

After 30 days of immersion, considered the quasi-saturation stage in the water uptake response, the friction behavior of all laminates is affected, although the severity still depends strongly on stacking sequence (Figure 4d). The B_8_ laminate again exhibits the highest COF, with µ increasing steadily from the running-in stage toward values around 0.6–0.7 and showing noticeable fluctuations, indicative of extensive interfacial damage, fiber protrusion, and unstable third-body flow in the fully saturated basalt-epoxy system. The B_2_C_4_B_2_ laminate follows a similar trend with a plateau near 0.45–0.5, remaining lower than B_8_ but significantly above the reference state, which reflects its lower but still substantial water uptake and the presence of basalt skins. In contrast to shorter aging times, the carbon-containing laminates now exhibit a clear increase in COF. The C_2_B_4_C_2_ laminate has an intermediate value increase in the approximate range 0.25–0.35, and the C_8_ has a steady-state value in the range 0.15–0.20. These increases reveal that prolonged exposure eventually overcomes the barrier effect of carbon fibers, leading to matrix microcracking and roughness amplification that enhance friction, although the absolute levels remain markedly lower than in the basalt-skinned configurations. Overall, Figure 4 demonstrates that pressurized water aging at 1 Hz first triggers a severe and early friction penalty in basalt-rich laminates and, at longer times, progressively degrades even the carbon-dominated systems, which underscores the critical influence of outer ply material and water uptake kinetics on the tribological durability of these hybrid laminates. This is because water immersion may induce significant effects on the tribological performance of fiber-reinforced composites due to the plasticization of the matrices with a consequent effect on the fiber/matrix interfaces. For instance, effect of hygrothermal aging on the mechanical and frictional wear properties of carbon fiber reinforced composites showed that for CFRP immersed in distilled water at 60 °C up to 90 days, the coefficient of friction (COF) dropped by up to 54% relative to unaged samples, whereas wear rate and wear scar width increased dramatically respectively up to 254.6% and 114.9% indicating severe interfacial degradation and matrix softening while water molecules act as internal lubricants [41]. Similarly, Özsoy et al. [23] reported a nonmonotonic COF evolution during aging; an initial decrease (as low as 20–40% below reference after 14 days) attributed to reorganization of a third-body tribofilm, followed by a partial recovery of friction with prolonged immersion, concomitant with increased wear scar width/depth due to fiber/matrix debonding and debris-assisted three-body abrasion. Walczak et al. [42] showed that water absorption in polymeric composites reduces cohesive strength and promotes fiber–matrix debonding; tribologically, this often manifests as increased wear rates and wider/deeper scars together with either transient reductions in COF (due to temporary water-lubrication or formation of a soft tribofilm) or later COF increases as third-body debris and fiber pull-out dominate.

**Figure 5:**
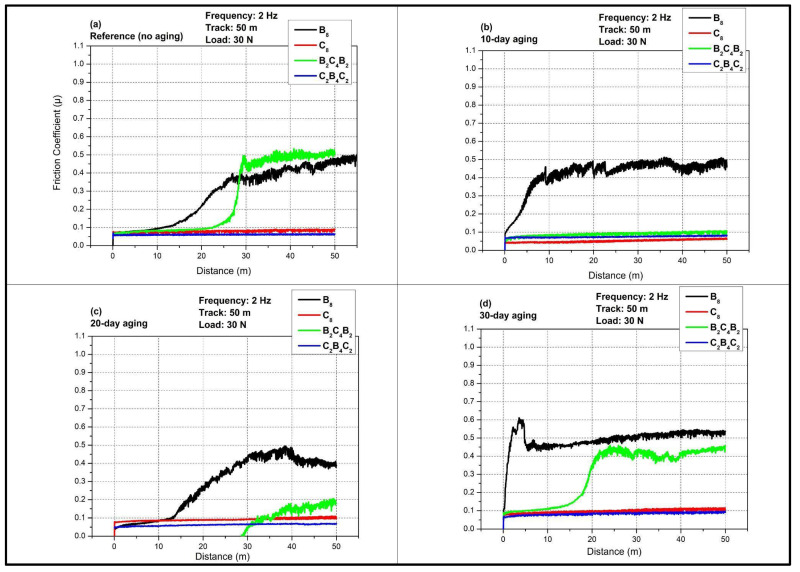
Effect of water aging time on COF values at 30 N load, 50 m track at 2 Hz in reciprocating wear tests: (**a**) Reference (no aging); (**b**) 10-day aging; (**c**) 20-day aging; (**d**) 30-day aging.

Figure 5 shows the evolution of the coefficient of friction (COF, µ) as a function of sliding distance for the four laminate configurations at 2 Hz under a 30 N normal load and a total reciprocating track length of 50 m for different pressurized water aging times. The curves again exhibit a short running-in regime followed by a quasi-steady state region, but the higher sliding frequency modifies both the absolute COF levels and their sensitivity to water immersion when compared with the 1 Hz condition. In general, increasing the frequency to 2 Hz leads to more stable and slightly lower steady state COF values, particularly for the basalt-dominated laminates, which suggests that the shorter reversal time limits severe stick–slip events and reduces the dwell time available for water-assisted debonding and microcrack propagation at the contact interface.

In the unaged reference condition in Figure 5a, all laminates start with a low COF of about 0.04–0.06 during the first meters of sliding. The C_8_ and C_2_B_4_C_2_ laminates maintain a nearly flat response across the entire 50 m track with steady state µ values below approximately 0.10, consistent with a mild adhesive or mixed adhesive and abrasive regime on relatively smooth carbon-rich surfaces. The B_8_ laminate displays a gradual increase in COF with distance, reaching a steady state level around 0.35–0.40 with moderate fluctuations, which reflects the somewhat rougher and more compliant basalt fiber surface and a higher propensity for ploughing and microfracture of the matrix close to the contact. The B_2_C_4_B_2_ laminate presents a distinct step-like transition. It remains at a low COF comparable to the carbon-based laminates up to approximately 25–30 m, then exhibits a rapid rise to a plateau near 0.50–0.55, indicating a breakthrough of the resin-rich top layer and exposure of the damage-prone basalt outer plies to direct contact with the steel counterbody.

After 10 days of water immersion, the frictional behavior in Figure 5b becomes more differentiated but remains less severe than at 1 Hz. The B_8_ laminate now shows an early increase in COF after the running-in stage, quickly reaching a plateau around 0.45–0.50 that is maintained with relatively small oscillations over the rest of the track. This behavior suggests that water-induced plasticization and interfacial weakening have increased the real contact area and facilitated mild micro ploughing, but the higher frequency prevents the large stick–slip excursions observed at 1 Hz. The B_2_C_4_B_2_ laminate exhibits only a modest rise in COF compared with the unaged state. Its curve stays below approximately 0.15–0.18 with a slight upward slope, indicating that the basalt skins are affected by water uptake yet retain a relatively stable tribological response at this aging level and frequency. This quasi-constant COF does not imply that the epoxy-rich near-surface film remains intact throughout the test. Rather, at 10-day ageing, the absorbed water primarily plasticizes the epoxy, increasing its ductility; under the higher sliding frequency (2 Hz), the sheared epoxy is removed more gradually and tends to smear and compact into a stable transfer layer on the steel counterbody and within the wear track. This ‘self-conditioned’ tribofilm stabilizes the real contact conditions and masks an abrupt matrix-to-fiber transition within the 50 m track length, because significant basalt-tow protrusion and extensive fiber-controlled ploughing are not yet dominant at this early ageing stage. Consequently, the COF rapidly reaches a steady state after the running-in period and remains nearly constant, whereas at longer aging times the progressive interfacial weakening and debris generation destabilize the friction response. The C_8_ and C_2_B_4_C_2_ laminates remain almost insensitive to 10-day aging and show low and very stable COF values similar to the reference condition, confirming again the barrier effect of carbon outer layers and their lower affinity for water absorption. With 20 days of water aging in Figure 5c, the B_8_ laminate still presents the highest COF among the four architectures, but the curve shape indicates a more gradual transition to steady state. After a short, low-friction start, µ increases progressively to approximately 0.45–0.50 and then fluctuates within a moderate band. This suggests that the near-surface matrix has evolved toward a homogeneous plasticized state, so that further damage accumulation results in a stable but relatively high friction plateau rather than a continuously rising trend. The B_2_C_4_B_2_ laminate remains at low COF levels for a substantial portion of the track, then experiences a sudden jump near 30–35 m up to about 0.15–0.20, which reveals delayed exposure of the inner plies and progressive removal of softened matrix and fiber fragments. The C_8_ and C_2_B_4_C_2_ laminates again display only minor changes. Their steady state COF values increase slightly but remain below approximately 0.10, with very limited noise, indicating that water-induced microcracking and roughness amplification are still modest at this intermediate aging time when sliding at 2 Hz.

After 30 days of immersion, considered the quasi-saturation condition, all laminates show some influence of water aging in Figure 5d, although stacking sequence effects remain pronounced. The B_8_ laminate exhibits an initial overshoot where µ rapidly rises to values close to 0.60 within the first few meters, followed by a relaxation toward a quasi-steady state around 0.45–0.50. This behavior points to a severely degraded and water-saturated near-surface region that is quickly sheared off during the early cycles, after which a more stable but still damaged subsurface controls friction. The B_2_C_4_B_2_ laminate shows a delayed but pronounced increase in COF. The curve remains low up to approximately 25–30 m, then climbs steeply to a plateau near 0.30–0.35, reflecting significant matrix plasticization and interfacial weakening in the basalt skins once water saturation has been achieved. The C_2_B_4_C_2_ laminate begins to show a clearer effect of prolonged aging, with the COF rising gradually to approximately 0.15–0.20 toward the end of the track, while the C_8_ laminate reaches a steady state µ of about 0.12–0.15, still the lowest among the configurations. Overall, Figure 5 demonstrates that at 2 Hz the carbon-based laminates preserve low and stable friction even after extended water exposure, whereas the basalt-dominated laminates remain more sensitive to water aging, although the severity and instability of their friction response are mitigated compared with the lower frequency condition. Dhieb et al. [43] demonstrated that while water immersion and high ambient humidity promote matrix plasticization and fiber–matrix interfacial degradation in CFRP, increasing the sliding rate suppresses adhesive locking by limiting moisture-assisted dwell time, thereby substantially stabilizing the friction trace under reciprocating motion. Walczak et al. [42] similarly reported that water-absorbed fiber-reinforced thermoplastic composites develop weakened interfacial adhesion and larger wear scars; however, higher oscillation speed reduces the time available for strong adhesive junctions to form, resulting in a transition from unstable stick–slip to a more uniform abrasive or mixed wear regime with comparatively smoother COF evolution. Talib et al. [44] emphasized that in fiber-reinforced polymer systems, dynamic contact conditions, especially high-frequency reciprocating sliding, shift the wear mechanism from intermittent adhesion-dominated interactions to fatigue-assisted micro-ploughing, which yields more stable and predictable friction behavior despite pre-existing environmental damage.

### 3.2. Wear Damage Evaluation

**Figure 6:**
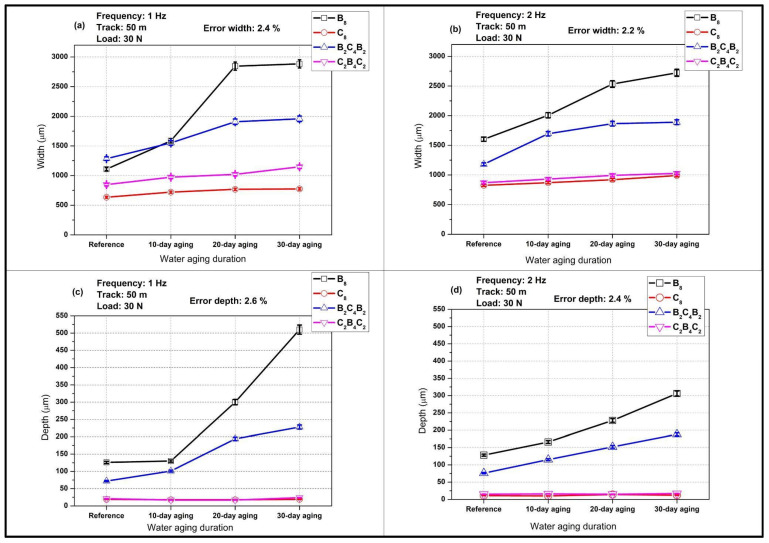
Comparison of width and depth values in reciprocating wear tests at 30 N load, 50 m track with various aging durations: (**a**) width comparison at 1 Hz; (**b**) width comparison at 2 Hz; (**c**) depth comparison at 1 Hz; (**d**) depth comparison at 2 Hz.

In Figure 6, the variation in width and depth of wear scars for different stacking orders for varying durations of water ageing when tested under a load of 30 N, at a track of 50 m, and at frequencies of 1 Hz and 2 Hz can be viewed. The data provide a quantitative measure of the material loss trends that were qualitatively inferred from the COF curves in Figure 4 and Figure 5. For both frequencies, increasing water exposure leads to wider and deeper wear tracks for all laminates, yet the rate of increase is strongly dependent on the outer ply material. Basalt-dominated laminates (B_8_ and B_2_C_4_B_2_) exhibit much larger widths and depths and a steeper growth with immersion time, whereas carbon-dominated configurations (C_8_ and C_2_B_4_C_2_) show only modest changes, confirming their superior resistance against pressurized water-induced degradation. The relatively low experimental errors for width and depth (about 2–3%) highlight the good repeatability of the measurements and the robustness of the observed trends.

In Figure 6a, showing a comparison of wear scar width at 1 Hz, it can be seen that large differences between the laminates are possible even in the unaged condition. The B_8_ laminate already shows the largest width, approximately 1100 µm, followed by B_2_C_4_B_2_ at about 1300 µm, while C_2_B_4_C_2_ and C_8_ remain near 800–900 µm and 600 µm, respectively. With 10 days of water aging, all widths increase, but the effect is most pronounced for the basalt-rich systems. The B_8_ width rises to roughly 1500 µm and B_2_C_4_B_2_ to about 1500 µm, whereas C_2_B_4_C_2_ and C_8_ increase only slightly by around 100 µm. After 20 days, B_8_ experiences an abrupt growth to nearly 2800 µm, indicating a transition to a much more severe wear regime that is consistent with the high COF levels recorded at this frequency. B_2_C_4_B_2_ registration shows 1900 micrometers, and carbon-based laminates retain values lower than 1100 micrometers. Upon reaching 30 days, B_8_ indicates about 3000 µm and B_2_C_4_B_2_ about 2000 µm, while C_2_B_4_C_2_ and C_8_ are at much lower values of about 1200 µm and 800 µm. These trends confirm that water-saturated basalt outer plies promote extensive material removal under low-frequency reciprocating loading.

Figure 6b presents the width evolution at 2 Hz and reveals that increasing the sliding frequency mitigates, but does not suppress, the detrimental influence of water aging, particularly for the basalt-dominated laminates. In the reference condition, the B_8_ width is already larger than at 1 Hz, around 1700–1800 µm, which suggests that the higher frequency enhances the initial removal of asperities and weak surface regions. However, the subsequent increase with aging time is more gradual than in Figure 6a. The B_8_ width grows to about 2200 µm after 10 days and to roughly 2500–2700 µm at 20-day and 30-day, showing a nearly linear trend instead of the abrupt jump observed at 1 Hz. The B_2_C_4_B_2_ laminate follows a similar but less pronounced pattern, rising from about 1200 µm in the reference state to approximately 1600 µm, 1800 µm, and 1900 µm at 10-day, 20-day, and 30-day. The carbon-based laminates again show the smallest widths and the weakest dependence on aging. C_8_ and C_2_B_4_C_2_ widths increase by only about 100–200 µm over the full immersion range and remain below 1100 µm, reflecting their superior dimensional stability and lower susceptibility to moisture-induced softening at higher sliding frequency.

Figure 6c focuses on wear scar depth at 1 Hz and further emphasizes the strong interaction between water aging and laminate architecture. In the reference condition, B_8_ has a depth of approximately 150 µm, more than twice that of B_2_C_4_B_2_ (about 80 µm) and an order of magnitude higher than C_8_ and C_2_B_4_C_2_, which remain near 10 µm and 20 µm. After 10 days, depths for all laminates increase only modestly, yet a pronounced acceleration is observed for the basalt-rich systems at longer aging durations. At 20 days, the B_8_ depth nearly doubles to approximately 300 µm, while B_2_C_4_B_2_ reaches about 170 µm, indicating a transition to deeper ploughing and subsurface cracking, in agreement with the high COF and unstable friction response. By 30 days, B_8_ attains a depth close to 500 µm, and B_2_C_4_B_2_ approaches 230 µm, showing that a fully saturated basalt epoxy interface is highly vulnerable to penetrative wear. In contrast, C_2_B_4_C_2_ and C_8_ show only small incremental increases across the entire aging range, and their final depths remain below roughly 50 µm and 20 µm, respectively, indicating that wear remains in a mild regime dominated by surface smoothing rather than bulk material removal.

Figure 6d compares the depths at 2 Hz and reveals that, similar to the width trends, higher frequency reduces the severity of wear, particularly for the basalt-dominated laminates, although the relative ranking of the materials remains unchanged. For B_8_, depth increases from approximately 150 µm in the reference state to about 180 µm, 220 µm, and 280–300 µm after 10-day, 20-day, and 30-day, respectively. The growth is nearly linear, without the abrupt jump observed at 1 Hz, which suggests that the reduced contact time per cycle limits the development of deep subsurface cracks and the formation of thick third-body layers. B_2_C_4_B_2_ shows a similar behavior, with depths rising from about 60 µm to approximately 80 µm, 120 µm, and 150 µm over the same immersion intervals. The carbon-based laminates again exhibit the smallest depths and the weakest sensitivity to aging, with C_2_B_4_C_2_ increasing only from about 20 µm to around 35–40 µm and C_8_ from roughly 10 µm to about 20 µm. Overall, Figure 6 demonstrates that water aging significantly amplifies wear scar dimensions, especially for basalt-skinned laminates, and that increasing the sliding frequency from 1 to 2 Hz helps to moderate both width and depth, although it cannot fully compensate for the detrimental effect of long-term pressurized water exposure in the basalt-dominated architectures.

**Figure 7:**
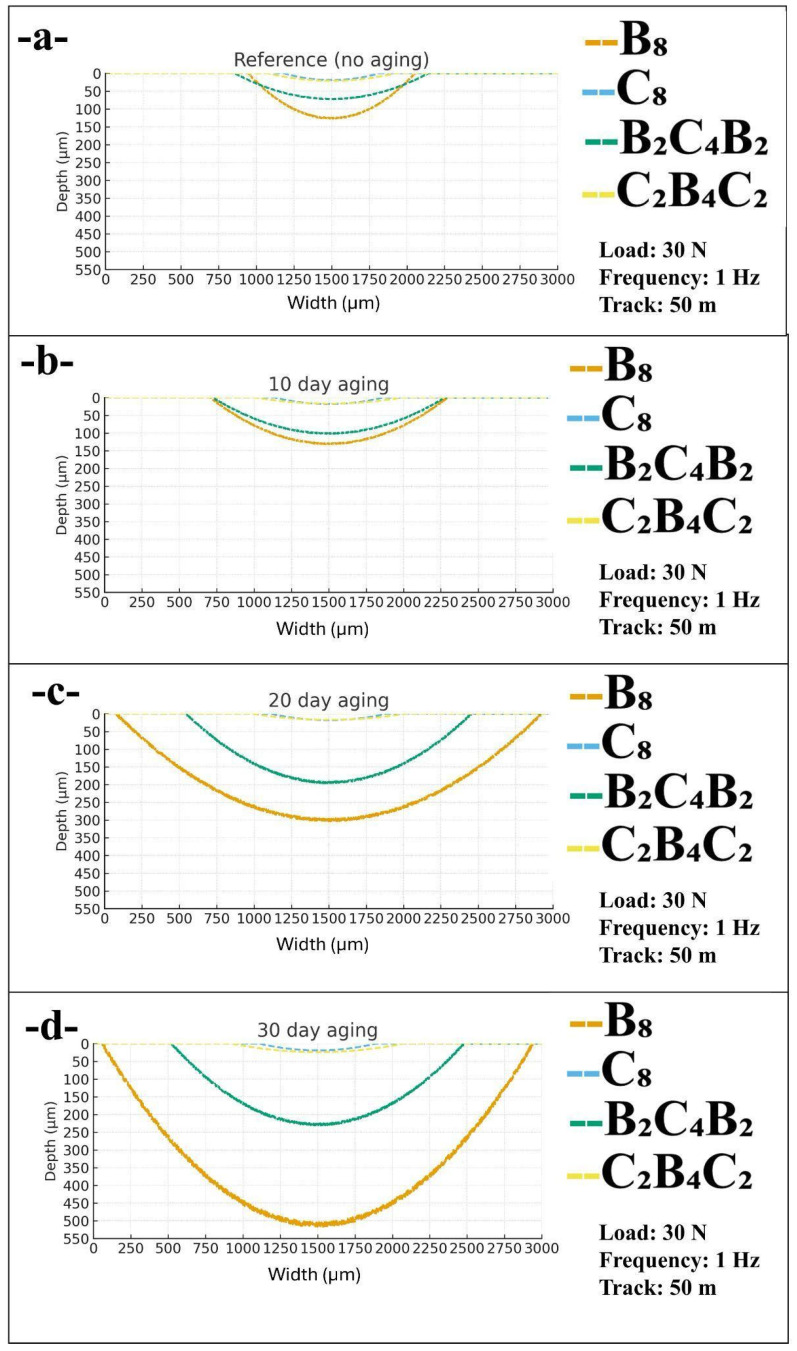
Comparison of width and depth values in reciprocating wear tests at 30 N load, 50 m track at 1 Hz with various aging durations: (**a**) reference (no aging); (**b**) 10-day aging; (**c**) 20-day aging; (**d**) 30-day aging.

Figure 7 compares the cross-sectional profiles of the reciprocating wear scars for all stacking sequences at 1 Hz, 30 N, and a 50 m track as a function of water aging time. Width and depth were extracted from the 3D profilometry data and plotted together to visualize both the lateral extent and the penetration of wear into the laminate surface. The parabolic shapes illustrate the typical bowl-like morphology generated by reciprocating sliding, while the relative position and curvature of each profile reveal how the combination of water uptake and outer-ply material controls the severity of material removal. Consistent with Figure 4 and Figure 6, the all-basalt laminate (B_8_) develops the widest and deepest scars, the basalt-skinned hybrid (B_2_C_4_B_2_) shows intermediate damage, and the carbon-dominated laminates (C_8_ and C_2_B_4_C_2_) retain narrow and shallow tracks even after prolonged pressurized water aging.

In the reference, unaged condition shown in Figure 7a, all profiles are relatively shallow and close to the surface, but clear differences between laminates are already evident. The B_8_ track displays the largest width, about 1108 µm, and a depth of 126 µm, indicating more extensive micro ploughing of the softer basalt-rich surface. In contrast, the B_2_C_4_B_2_ hybrid has a slightly wider and much less deep bowl, with width and depth measurements of 1285 µm and 72 µm, respectively, attributing this to the effect of the inner carbon plies on load carrying and arresting the propagation of cracks. In contrast, the C_2_B_4_C_2_ and C_8_ laminates have very narrow and less deep bowls, measuring 847 µm and 634 µm in width and 21 µm and 19 µm in depth, respectively. These results reaffirm that un-aged carbon skins can adequately resist both in-plane and out-of-plane movements of wear.

After the 10 days of immersion in water, the profiles in Figure 7b slightly broaden, and the values are small for the majority of the laminates. In this case, the width of the B8 laminate increases to 1587 μm, and the maximum depth marginally increases to 130 μm. In addition, this indicates that the uptake of water increases the contact area due to softening of the near-surface material. Further, the B_2_C_4_B_2_ laminate widens to 1551 μm in width and reaches a depth of 101 μm, showing that ploughing action slightly increases due to the absorption of water in the basalt skins. In carbon-based laminates, there is little change in width and depth for C_2_B_4_C_2_ and C_8_ laminates, measuring 973 μm, 17 μm in width and depth, respectively, and for C_8_, 720 μm and 18 μm in width and depth, respectively, showing that the values are within the experimental errors and are similar to those in the reference state. In this case, it can be concluded that only small increases in dimensions of the wear scar are observed after short-term aging, and the ranking of the laminates also remains similar.

From 20 days of exposure, in the radial bow of different specimens (Figure 7c), it can be seen that a considerable disparity in behavior exists between basalt and carbon configurations. In the case of B_8_, the width and depth of the B8 profile are 2845 μm and 300 μm, respectively, and for hybrid specimens (B_2_C_4_B_2_), it can be seen that considerable damage has taken place, denoted by a width of 1908 μm and depth of 194 μm. Even in B_2_C_4_B_2_, it can be observed that the damage is less compared to that in B8 basalt specimens, and it can be seen that considerable basalt outers’ effect exists at the point when the amount of absorbed-water content approaches saturation limit values in the specimen. In contrast, for the C_2_B_4_C_2_ and C_8_ specimens, it can be observed that very shallow bowls are obtained, denoted by the width of 1020 μm and 768 μm, and depth of about 20 μm and 18 μm, respectively.

After 30 days of immersion, corresponding to the highest aging level in this study, the differences shown in Figure 7d become even more pronounced. The B_8_ laminate exhibits an extremely wide and deep scar, with a width of 2883 µm and a depth of 510 µm, indicating catastrophic local degradation in which the wear process has penetrated far into the laminate thickness. The B_2_C_4_B_2_ hybrid also experiences a further increase to 1957 µm in width and 228 µm in depth, confirming that long-term water saturation of basalt skins substantially compromises wear resistance even when carbon plies are present in the interior. The C_2_B_4_C_2_ laminate shows only a moderate widening to 1147 µm and a slight depth increase to 24 µm, while the C_8_ laminate remains the most resistant, with a width of 774 µm and depth of 19 µm that are only marginally higher than the reference values. Collectively, the profiles in Figure 7 demonstrate that at 1 Hz, the combination of basalt outer plies and prolonged pressurized water aging results in dramatic increases in both width and depth of the wear tracks, whereas carbon-skinned laminates maintain narrow and shallow scars, underscoring their superior tribological durability in humid service environments.

**Figure 8:**
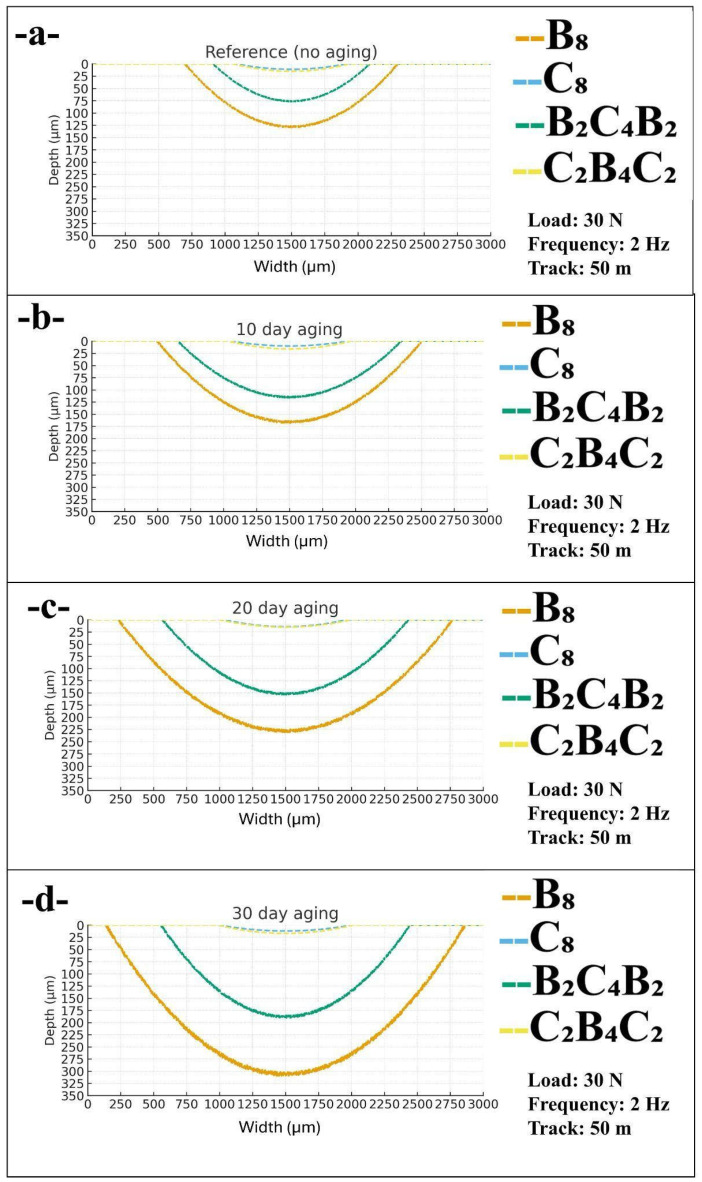
Comparison of width and depth values in reciprocating wear tests at 30 N load, 50 m track at 2 Hz with various aging durations: (**a**) reference (no aging); (**b**) 10-day aging; (**c**) 20-day aging; (**d**) 30-day aging.

Figure 8 compares the cross-sectional wear scar profiles obtained at 2 Hz, 30 N, and a 50 m reciprocating track for different pressurized water aging durations. The plots show the parabolic shape of the scars in terms of width versus depth, allowing a direct visual comparison of the lateral spread and penetration of wear for each stacking sequence under higher sliding frequency. Similar to the 1 Hz case, the all-basalt laminate B_8_ develops the largest and deepest bowls, the basalt-skinned hybrid B_2_C_4_B_2_ shows intermediate damage, and the carbon-dominated C_8_ and C_2_B_4_C_2_ laminates retain narrow and shallow scars. However, the maximum depths at 2 Hz remain lower than those at 1 Hz for the same aging level, which indicates that the shorter contact time per cycle mitigates severe subsurface damage even in the water-saturated basalt-rich systems.

In the reference, non-aged condition (Figure 8a), all laminates exhibit relatively shallow profiles, but clear differences in scar dimensions are already present. In B_8_ laminate, the bowl is widest and deepest, measuring about 1602 µm in width and 128 µm in depth, signifying higher levels of micro-ploughing in the basalt-containing layering. Conversely, in B_2_C_4_B_2_ hybrid laminate, it is narrower and less deep, measuring about 1180 µm in width and 76 µm in depth, signifying that the inner layer of carbon plies serves as an advantage in resisting the penetration process. The C_2_B_4_C_2_ and C_8_ laminates have the smallest scars, with widths of 870 and 825 µm and depths of only 15 and 11 µm, respectively, which confirms that carbon skins provide an intrinsically higher resistance against reciprocating wear at 2 Hz even before water exposure.

In the context of 10 days of water aging (Figure 8b), it can be seen that the specimens undergo a broadening and deepening of the profiles for all specimens, and this effect is more evident in those enriched with basalt. In specimen B_8_, the area and depth of the scar increase to 2007 µm and 166 µm, respectively, denoting an increased softening of the matrix and decreased binding between the materials, making it easier for the counterbody to indent. In the case of hybrid B_2_C_4_B_2_, similar effects are obtained, denoting an increased width and depth of 1695 µm and 115 µm, respectively. In contrast, the carbon-dominated laminates experience little sensitivity, denoting that the width and depth of the scar marginally increase to 930 µm and 16 µm, and 870 µm and 13 µm for specimens C_2_B_4_C_2_ and C_8_, respectively.

By 20 days of exposure (Figure 8c), the difference in wear between basalt and carbon laminates starts to be evident. In the B_8_ laminate, the profile widens to 2534 µm and deepens to 228 µm, showing increased microcracking, fiber-matrix separations, and removal of the softened material from the near-surface zone. In the B_2_C_4_B_2_ laminate, the width and depth are 1865 µm and 152 µm, respectively, confirming that saturation of the basalt laminate increased the severity of wear, but the carbon materials still suppressed the final penetration depth compared to the B8 laminate. In contrast, in the C_2_B_4_C_2_ and C_8_ laminates, the profiles remain relatively steady. The width increases slightly to 993 µm and 920 µm, and the depth remains at about 15 µm and 14 µm, respectively, indicating that the wear process for these carbon-based laminates remains mainly gentle and leads to only very small material removal or just surface polishing.

Based on the 30-day immersion period that equates to the highest level of aging, the damage characteristics emerging in the profiles of Figure 8d indicate, for 2 Hz frequencies, the augmented effect of exposure to water. In this case, the damage in the B_8_ laminate is the worst, featuring 2723 µm width and 306 µm depth of the scar, signifying that it has caused substantial damage to the thickness and, more importantly, that the basalt-epoxy interfaces are particularly susceptible to damage due to exposure to water. In the case of hybrid B_2_C_4_B_2_, it can be noticed that the width and depth of damage are increased to 1890 µm and 188 µm, respectively, emphasizing that in the case of basalt skins, despite the addition of carbon in the internal structure, it essentially remains the leader in determining the resistance of materials towards wear-off. In contrast, in the carbon-dominated laminates, it can be seen that in comparison to the unaged state, the width and depth of the scar are only marginally increased in both the C_2_B_4_C_2_ laminate to 1027 µm and 17 µm, and in the C_8_ laminate to 990 µm and 12 µm, respectively, signifying that in comparison to the basalt-based materials, the carbon-based materials have better resistance to wear-off properties when exposed to humid environments during corroborated and rapid sliding conditions.

This increase in wear scar width and depth with increasing duration of water immersion, as shown in Figure 6, Figure 7 and Figure 8, agrees with degradation kinetics reported in literature for fiber-reinforced polymers exposed to environmental immersion. Moisture diffusing into epoxy compacts the material, thus reducing cross-linking, with consequential effects on reduced hardness that promotes greater ploughing as a result of sliding [45]. Sang et al. [45] were able to show that hydrothermal degradation results in the softening of near-surface layers, thus increasing wear scar width as well as wear scar depth with increasing cycles as a result of sliding. Consequent softening of the bulk material, in addition, results in greater concentration of water along the fiber-matrix interfaces, so as to increase debonding that thus further opens pathways for scar width increase. Indeed, this is supported further by the work of Wong et al. [46], as water absorption was shown significantly to degrade the mode II interlaminar fracture toughness properties of carbon fiber laminates, thus as a result opening pathway for easier initiation as well as propagation along paths that are lateral with respect to the direction of sliding, as was determined in this work. Indeed, further studies showing sliding properties of hydrothermally degraded polymeric composites would further indicate that water changes the mode of wear from being abrasive-cutting wear, with faster rates of formed debris, as was evidenced by Belotti et al. [21]. Such changes provide further explanations for the increase in scar width as a result of immersion, as shown in Figure 6, Figure 7 and Figure 8. Indeed, further evidence showing degradation through higher water absorption was given further support through modeling studies that would show higher degradation along fiber-matrix interfaces as a result of higher affinity with water, thus, as a result, providing pathways for higher degradation with subsequent increase in wear scar dimensions, as shown in this work. Indeed, further studies showing effects along fiber-matrix interfaces were shown in the reduced friction coefficients, as was illustrated by Song et al. [47], who further indicated degradation in greater wear groove depth as well as scar dimensions as a result of degradation.

### 3.3. Wear Track SEM Damage Analysis

**Figure 9:**
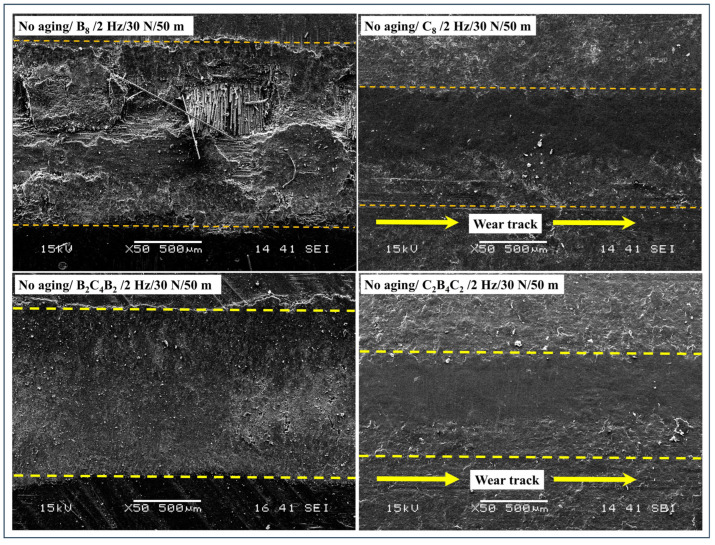
SEM Wear Track Morphologies of Basalt/Carbon Hybrid Laminates under No-Aging Condition (2 Hz, 30 N, 50 m).

Under the non-aging variant (2 Hz, 30 N, 50 μm), marked variations of wear-damage topographies were observed based on the stacking sequence (Figure 9). In the case of B_8_, extensive abrasive wear took place, including sharp micro-ploughing grooves, intense matrix transfer, fiber protrusion, and micro-damage to fibers, reflecting high wear track width and height, and high and stable friction coefficients (COF). In contrast, C_8_ samples presented the lowest level of contact surface deterioration, including smooth-abraded contact areas, with insignificant micro-scratching, minor tribo-film formation, and negligible micro-damage to fibers, corresponding to narrow wear tracks and small wear depths, along with the lowest COF. In the case of the B_2_C_4_B_2_ and C_2_B_4_C_2_ hybrid laminates, intermediate tribological performance occurred, involving moderate wear topographies characterized by micro-ploughing generated by top basalt layers, along with suppression of subsurface micro-damages by carbon fibers, including average wear tracks and average COF, sandwiched between the corresponding B_8_ and C_8_ extremes. In contrast, specimens of C_2_B_4_C_2_ laminate were characterized by very similar tribological properties and near-identical wear topographies, including smooth surfaces with very few micro-grooves and interfacial micro-debonding, reflecting narrow wear tracks and low wear depths, and exhibiting validation regarding inferior subsurface micro-damages. In the aggregate, the unaged samples clearly show the transition of wear modes, predominantly shifting to mild contact ‘adhesive-polishing’ by basalt- and carbon-skinned tribosystems.

**Figure 10:**
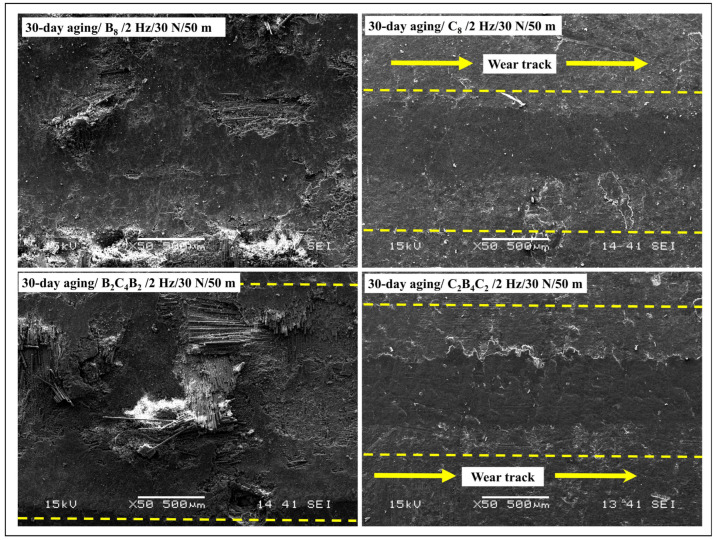
SEM Wear Track Morphologies of Basalt/Carbon Hybrid Laminates after 30-Day Pressurized Water Aging (2 Hz, 30 N, 50 m).

After 30 days of pressurized water immersion, aging effects notably intensified the wear damage, particularly in laminates with basalt as the outer ply (Figure 10). The B_8_ laminate displayed extensive fiber–matrix debonding, widespread fiber pull-out, fractured fiber ends, deep ploughing grooves, and heavy third-body debris accumulation, indicating a transition from pure abrasive wear toward a combined abrasive–fatigue wear mechanism. These microstructural features directly correlate with the sharply increased wear track width and depth, as well as the unstable and elevated COF behavior recorded after aging. In the case of C_8_, minor increments of surface roughness and micro-scratches were found, but the carbon fiber skin supported the structure, reducing the rate of matrix deterioration and interfacial damage. As such, the dimensions of the wear tracks were small, and the increment of the Coefficient of Friction (COF) was negligible. In a hybrid of B_2_C_4_B_2_, extensive deterioration of the basalt surfaces, characterized by high levels of fiber pull-out, micro-cracking, and nascent stages of delamination, occurred, but was partly resisted by the carbon layers, which reduced the rate of cracks propagating towards the laminate interior. This resulted in high wear depths and widths greater than the unaged laminate, but were, again, lower than B_8_. In contrast, the C_2_B_4_C_2_ hybrid laminate sustained the most stable morphology among aged hybrids, displaying mainly fine micro-scratches and limited local debonding without widespread fiber fracture or delamination. Accordingly, its wear geometry and COF increases remained modest after aging, confirming that a carbon outer layer effectively acts as a diffusion barrier against water ingress and preserves tribological stability under reciprocating sliding conditions. The distinct, baseline wear morphologies observed under no-aging conditions closely match previously reported tribological behavior of basalt- and carbon-reinforced epoxy systems. Specifically, basalt-rich surfaces tend to promote abrasive micro-ploughing and localized matrix fracture due to their rougher fiber topography and lower near-surface stiffness, whereas carbon-skinned architectures present smooth, polishing-dominated worn surfaces with limited fiber fracture and low steady COF because of the high stiffness and surface smoothness of carbon plies [48]. Oliveira et al. [49] directly link water immersion to epoxy plasticization, the formation of microfibrillar/porous regions, and clear fiber–matrix debonding visible by SEM, which in turn increases real contact area and third-body generation under sliding. Rocha et al. [50] demonstrate that hygrothermal ingress promotes interfacial weakening and severe mechanical property losses that accelerate subsurface cracking and delamination under cyclic loading. Choi and Douglas [51] explain the thermomechanical fingerprint of such processes by documenting how water can both plasticize the matrix (lower apparent Tg) and create heterogeneous domains that intensify localized damage. A behavior that rationalizes the larger, unstable COF traces and the abrupt increases in wear track width and depth recorded for basalt-skinned architectures after long immersion.

### 3.4. DSC Analyses

**Figure 11:**
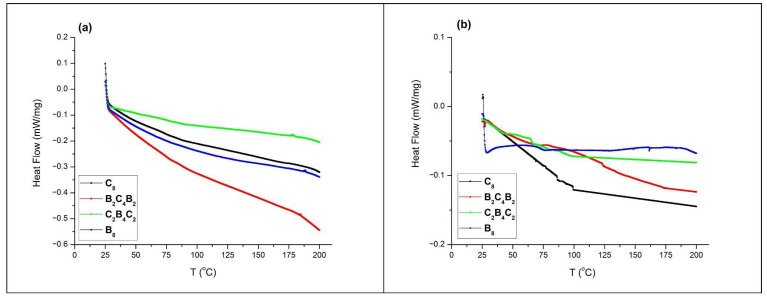
DSC curves of (**a**) unaged and (**b**) water immersion-aged hybrid composites after 30 days.

Figure 11 highlights the differential scanning calorimetry (DSC) scans of the hybrid composite laminates prior to aging (Figure 11a) and after 30 days of immersion under high-pressure water (Figure 11b) for the four respective configurations: B_8_, C_8_, B_2_C_4_B_2_, and C_2_B_4_C_2_. Prior to aging, it is important to note that each hybrid laminate exhibits a distinct and sharp transition corresponding to the nominal Tg interval between 90–95 °C, which is characteristic of the Tg value associated with the respective Sika CR80/CH80-2 hybrid matrix system. The sharp transition zone associated with each of the laminates, indicative of high homogeneity and cross-link density, is due to the adopted vacuum infusion process and post-curing. Comparing the un-aged laminates (Figure 11a), it is noticed that the carbon-containing surface topographies (C_8_ and C_2_B_4_C_2_) have a higher Tg value and more pronounced transition slope than the basalt-containing ones (B_8_ and B_2_C_4_B_2_). This is because carbon fibers have a lower affinity to moisture and are more inert to the chemical environment on the fiber surface. As such, carbon-containing samples are likely to have a more homogeneously characterized cross-link density near the top layer. Moreover, basalt-containing samples are likely to present residual traces of moisture after conditioning, and basalt fibers are also more hydrophilic, which could result in interfacial layers, increasing the width of the glass transition. After 30 days of pressurized water immersion aging (Figure 11b), it is obvious that each laminate configuration has a marked shift of the glass transition region towards lower temperatures, signifying plasticization of the epoxy matrix due to the presence of adsorbed water molecules. Water, therefore, acts as a secondary plasticizer, which interferes with intermolecular hydrogen bonding, thereby decreasing the effective cross-link density and, consequently, the Tg. Of particular interest is the observed fact that the depression of Tg and the width of the region associated with the glass transition are highly dependent on the stacking sequences. The most exaggerated depression and widening are found to occur in the B_8_ and B_2_C_4_B_2_ laminates, which correspond to the highest reported moisture contents in Figure 3.

The widening of the glass transition region reflects the onset of spatially heterogeneous plasticization, where the plasticization of the resin layers near hydrophilic basalt fibers occurs to a greater extent than the plasticization of the more remotely located layers. This is also reflecting the characteristic of non-uniform moisture diffusion and interfacially associated weakening, associated with micro-scale gradients of cross-link density and polymer chain mobility. Exactly the same trend of moisture-triggered thermal degradation has also been found by Mu et al. [52], who studied the continuous flax fiber-reinforced epoxy materials and reported a clear trend of Tg depression associated with the increase of the extent of hygro-thermal aging. Exactly the same trend of Tg depression, accompanied by a marked widening of the Glass transition region, has also been reported by Mu et al. [52] and reflects the onset of micro-scale, heterogeneously associated increments of molecular mobility, triggered by micro-scale, unequally distributed moisture. An analogous mechanism has also been reported by da Silva et al. [53], who noted an initially elevated Tg value following thermal curing, accompanied by post-curing increments of Tg, following which interfacially associated plasticization and relaxation triggered by exposure to moisture has been noted, and has been found to occur to a greater extent when exposure is associated with increased accessibility of moisture to interfacial, and greater amounts of un-aged, mature resin. In the case of the hybrid basalt carbon laminates, clearly the samples are completely self-consistent, where un-aged samples are characterized by Tg values reflecting the mature, initially un-aged, cured-epoxy systems, while the 30-day water-pressurized immersed samples are characterized by marked Tg depression and widening, reflecting interfacially associated plasticization.

### 3.5. Factorial Analysis

Factorial analyses are critical for understanding the behavior of multi-parameter processes in engineering applications. This approach not only reveals the influence of individual variables but also makes visible how multiple factors operating simultaneously in the system interact with each other. The overall factorial regression results reported in this study reveal the complex structure of the parameters affecting width, in particular, with a very high level of explanatory power. In this context, Table 2 provides a highly illustrative demonstration of the extent to which wear scar width is determined by which parameters. The fact that the model explains 99.95 percent of the total variance confirms that the factorial structure still offers high discriminatory power despite the large number of experiments. Table 2 shows that the most dominant main effect is the “material” factor. This finding is consistent with the experimental results in the article; This is because it has been clearly demonstrated in previous sections that basalt-based configurations (especially B_8_ and B_2_C_4_B_2_) exhibit significant width increases after exposure to water, whereas carbon-based structures (C_8_ and C_2_B_4_C_2_) exhibit much more limited growth. Therefore, the material factor contributing over 70 percent quantitatively confirms that the width response is directly related to the fiber architecture and water uptake tendency.

According to Table 2, another strong main effect is the time factor. This result is consistent with the profilometry analyses reported in the article; it was noted that the wear scar of basalt-based laminates widened suddenly and sharply, particularly on days 20 and 30, while carbon-based laminates exhibited a more limited change. Therefore, the high F-value of time statistically supports the experimental observation that matrix plasticization and interface weakening, which progress with water absorption, rapidly increase the width. Although the contribution of the main effect of frequency is relatively small, its significance in Table 2 is significant. This finding is consistent with the differences between 1 Hz and 2 Hz discussed in the relevant sections of the article. It has been experimentally demonstrated that more severe wear occurs at low frequency and a somewhat more stable contact condition occurs at high frequency; the statistical significance of frequency indicates that this behavior has a small-scale but consistent effect on width.

One of the most striking points in Table 2 is the significant findings of both the two-way and three-way interactions. The particularly high contribution of the material × time interaction quantitatively confirms that water uptake behavior creates a completely different wear regime depending on the material type. Although damage, especially that produced by water, increases markedly with basalt dominance, the stronger limited effect of time for carbon dominance explains the interaction. The presence of the significant three-way interaction indicates that frequency, especially, impacts the material and time interaction in a distinct but constrained fashion.

**Figure 12:**
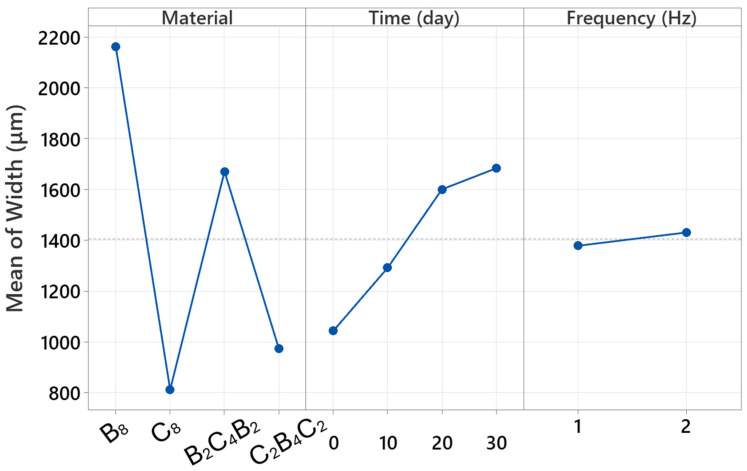
Factorial main effect diagram showing the average effects of material, time, and frequency on wear scar width.

The mean effect diagram for width, shown in Figure 12, illustrates well the impact of each important factor on the wear scar width. The trend of Figure 12 is well supported by the experimental observations reported in this study, and it suggests that the width of the wear scar is dependent on the material and time, but it is also affected by the frequency. According to the corresponding figure, the highest mean width values are observed in laminates with basalt skins, with the B_8_ and B_2_C_4_B_2_ configurations being particularly distinct from the others. This is directly related to water uptake behavior, as basalt surfaces induce faster plasticization and interlaminar weakening at the matrix-fiber interface, resulting in a rapid increase in width. In contrast, the line is significantly lower in C_8_ and C_2_B_4_C_2_ laminates with carbon skins, confirming that carbon limits wear scarring due to its lower water absorption and more stable surface properties.

In the part dealing with the temporal effect, it is seen that the mean width values exhibit a strong increase, and this is most evident on days 20 and 30. The increase shown in the graph is very close to the sudden width increments detected during the profilometry analysis of the article on the matrix plasticity and water diffusion phase. The frequency effect, although small, is regular. Width values are high at 1 Hz, and a slight decrease is noted at 2 Hz, which is in line with the experimental result obtained in the study, that a shorter contact time delays microcrack increment and third-body creation.

Table 3 clearly demonstrates which factors are dominant in determining wear scar depth. The model explains 99.99 percent of the variance, showing that depth is almost entirely driven by material, time, and frequency. The material factor has the strongest effect; its explanation of 64.5 percent of the total variation in Table 3 is directly consistent with the large depth differences observed experimentally. For example, the profilometry results show that the B_8_ laminate shows a depth of approximately 300 µm on day 30, while C_8_ only shows a depth of around 20 µm, which is the physical equivalent of this effect. The time factor also makes a significant contribution, explaining 12.8 percent of the total variance. This result is statistically significant the sharp increase in depth in the B_8_ and B_2_C_4_B_2_ laminates as water absorption increases rapidly between days 20 and 30. The frequency effect, although less pronounced, is significant; The 0.88% contribution and high F-value in Table 3 explain why the depth at 1 Hz increases to 500 µm towards day 30, while it remains around 300 µm under the same conditions at 2 Hz. Furthermore, the significant two-way and three-way interactions (especially material × time) indicate that depth increases exponentially with water advancement in basalt-based laminates, and this increase is modulated by frequency conditions.

**Figure 13:**
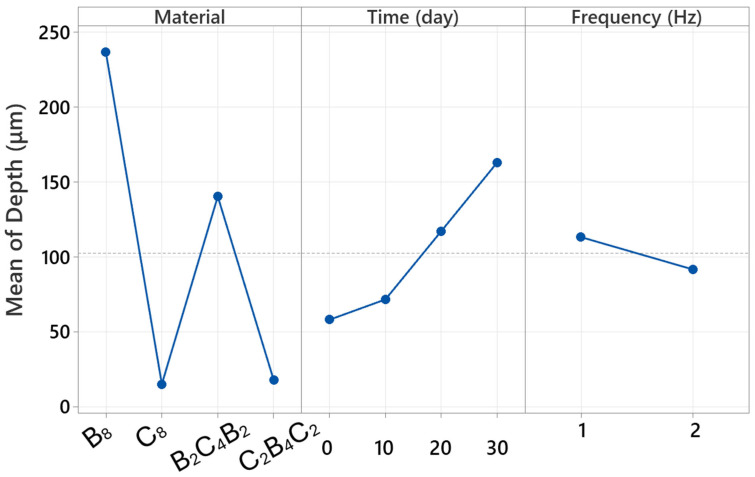
Factorial main effect diagram showing the average effects of material, time, and frequency on wear scar depth.

In Figure 13, it is evident that the material has the most significant effect on the average value of depth. The presence of basalt materials significantly increases the average value, and the average value of carbon materials is low. Although the trend is restricted, the increase in the average value of depth at 1 Hz compared to 2 Hz suggests the influence of contact strength. From the above, it is evident that the average value of depth is influenced by the material and time, and frequency is the secondary modulating factor.

Table 4 below highlights, in a detailed and systematic fashion, the factors influencing the Coefficient of Friction (COF). The factors influence a large amount of variance and show a high sensitivity of the COF to material architecture and contact. The material factor has the most influence, which is expected to agree with the experimental observations noted in the study. Carbon skin-containing laminates present lower amounts of COF, and basalt-containing ones show higher amounts of COF due to faster deterioration of the contacting surface. For example, although the COF remains more or less at 0.12 levels for the C8 structure, it increases to about 0.25 levels with progressive contact in B8 configurations. This difference clearly establishes the material influence. The time factor portrays a mild but significant influence on COF. With increased exposure to water, the aggregate level of surface deterioration due to basalt-containing laminates shows a mild increase in COF levels, which is most evident at 20 and 30 days, but is negligible for carbon skin-containing materials.

Frequency factor is also found to be a significant factor in the model, which gives credibility to the role of contact frequency on the coefficient of friction (COF). When the frequency is low, due to the increased contact time, micro surface tearing is increased, and consequently, the value of COF is moderately increased. On the contrary, when the frequency is high, which is accompanied by a low contact time, it causes a slight decrease in COF. As can be inferred from Table 4, interaction terms are found to obtain significance, which suggests that the role of COF is not determined by individual variables but is also affected by the interaction factor of material and frequency. The steeper increase of COF with low frequencies, evident for basalt-based composites compared to carbon-based ones, reflects such interaction.

**Figure 14:**
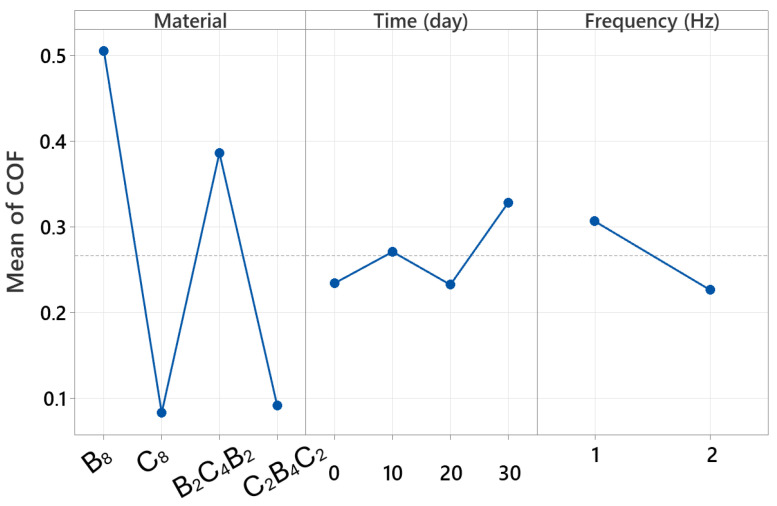
Main effect diagram showing the average response of the friction coefficient to material, time, and frequency factors.

Figure 14 shows a clear divergence in the material factor. Laminates with carbon-based outer surfaces produce the lowest COF values, while basalt-based laminates achieve higher average COF levels. This can be explained by the earlier onset of matrix softening and micro-surface fragmentation due to water absorption on basalt surfaces. Therefore, the distinct vertical separation between the material curves indicates that the primary determinant of friction behavior is the surface architecture.

The time-dependent effect, although of a smaller magnitude, reflects a moderate increase in the coefficient of friction (COF) with increasing exposure times. This is most apparent in the basalt-enriched laminates and reflects well the extent of surface deterioration found between days 20 and 30, as reported by the source article. In contrast, the carbon-based materials reflect very few changes with time, and this result clearly suggests high COF stability. In relation to the frequency factor, the COF is high at low frequencies but decreases moderately at higher frequencies. This may be explained by increased contact time at low frequencies, which are conducive to more extensive micro-tearing and fragmentation. Although the frequency factor is not very significant, it clearly suggests that the COF is more a function of the material itself, but is also indirectly affected by contact mechanics.

**Figure 15:**
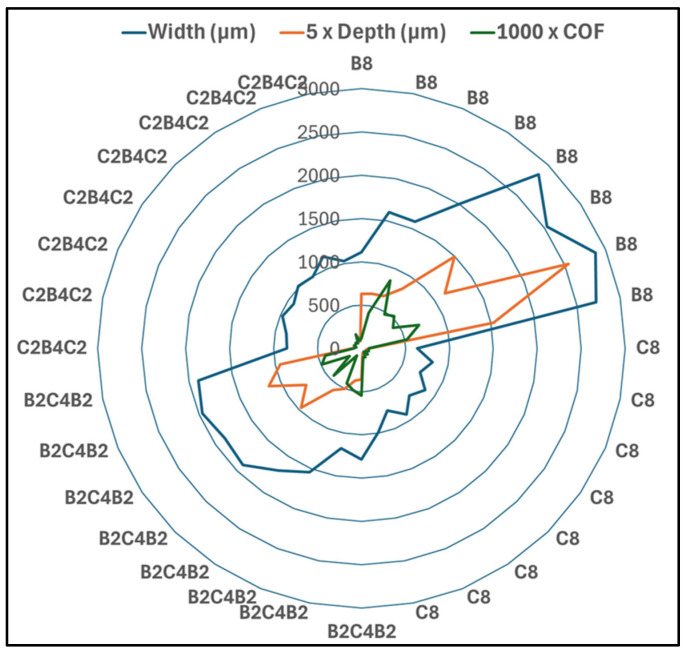
Radar diagram showing the tribological performance comparison of basalt, hybrid, and carbon-based composite laminates with respect to width, depth, and COF parameters.

In Figure 15, a radial chart is provided to enable a comparison among the tribological properties of four laminate designs (B_8_, B_2_C_4_B_2_, C_2_B_4_C_2_, and C_8_) on the same graphic. In it, the integration of the width, depth, and COF reported in the article allows, through a graphic representation, the assessment of the wear rate of the above-mentioned architectures when treated with water. The largest areas in Figure 15 belong to the basalt-dominated configurations. B_8_ protrudes significantly, particularly along the width and depth axes, consistent with severe water absorption, interfacial weakening, and progressive matrix plasticization between days 20 and 30 in the article. The higher magnitude of B_8_ on the COF axis also confirms that the surface integrity deteriorates more rapidly as the contact progresses.

When it came to the hybrid design B_2_C_4_B_2_, it resulted in a smaller radar field than with the B_8_ hybrid design, but it is still very broad. This clearly suggests that the hybrid design partly counteracts water sensitivity when it comes to basalt, although having basalt on the external layer resulted in a wear rate that is generally weak, and more so when compared to carbon-based designs. Laminates C_2_B_4_C_2_ and, more importantly, C_8_ represented the smallest areas on the radar chart. As a result of their narrow width and depth, and also owing to low COF, laminates C_2_B_4_C_2_ and, more importantly, C_8_ were found to be closer to the middle of the plot. These observations clearly support the fact that carbon’s external layer, which is evident in literature, offered benefits when it came to low water absorption, interfacial stability, and strong matrix and fiber interaction. Of importance to note is the fact that the C_8_ design clearly offered the most stable tribological performance when exposed to water and friction.

## 4. Conclusions

The present study demonstrates that both stacking sequence and pressurized water-immersion aging exert a pronounced and coupled influence on the reciprocating wear response of basalt, carbon, and hybrid laminates. Basalt-skinned configurations absorb the highest amount of moisture, reaching 4.3% at 30 days, whereas carbon-based and carbon-skinned hybrids remain in the 1.2–2.7% range, reflecting their lower permeability and more effective barrier behavior. Under 1 Hz sliding at 30 N and 50 m, this translates into a drastic widening and deepening of the wear scars for the B8 laminate, whose width and depth increase to about 2883 µm and 510 µm after 30 days, while C_8_ maintains narrow, shallow scars of approximately 774 µm and 19 µm, respectively. Increasing the sliding frequency to 2 Hz stabilizes friction and partially mitigates wear in basalt-rich laminates but does not alter the ranking dictated by the outer ply material, confirming that tribological durability in humid conditions is primarily governed by architecture rather than test frequency.

The multimodal microstructural and thermal analyses provide a consistent mechanistic explanation for these macroscopic trends. SEM observations reveal a transition from relatively mild abrasive or polishing wear in the unaged state to extensive fiber–matrix debonding, fiber pull out, third body accumulation, and mixed abrasive–fatigue mechanisms in basalt-skinned laminates after long-term pressurized immersion, whereas carbon-skinned laminates preserve comparatively smooth tracks with limited local damage. DSC measurements show that all laminates experience a downward shift and broadening of the glass transition region after 30 days at 10 bars, but that this plasticization effect is more severe in basalt-containing surfaces, consistent with their higher water uptake and more hydrophilic character. Non-contact profilometry further quantifies the evolution of wear scar geometry and confirms that the combination of basalt outer plies and high moisture content leads to a step change in wear depth and width once the saturation regime is approached.

From a design perspective, the radar map and main effects analysis synthesizing width, depth, and COF responses clearly show that basalt-dominated architectures occupy the largest damage envelope, while the C_2_B_4_C_2_ hybrid and especially all carbon C_8_ laminate cluster near the center of the diagram, with the smallest integrated wear footprint and the lowest and most stable friction. This evidence shows that placing carbon plies at the outer skin is an effective strategy to suppress water-driven degradation of tribological performance in marine or humid service conditions, while hybridizing with basalt in the interior allows partial retention of sustainability and cost benefits without sacrificing surface durability. Beyond these specific material choices, the study offers an original experimental framework that couples pressurized water-immersion aging at service-relevant pressure, frequency-dependent reciprocating wear testing, and correlated DSC, profilometry, and SEM analysis. This integrated approach can be extended to other hybrid composite systems to establish architecture-level design maps for tribological applications under combined mechanical and hygrothermal loading.

## Figures and Tables

**Table 1:** Reciprocating wear test parameters for hybrid composites after reference (no aging), 10-day, 20-day, and 30-day water immersion.

Hybrid Composite Type	Load	Reciprocating Wear Test Track	Wear Test Frequency	Water Aging Duration
B_8_ C_8_ B_2_C_4_B_2_ C_2_B_4_C_2_	30 N	50 m	1 Hz 2 Hz	Reference (no aging) 10-day 20-day 30-day

**Table 2:** General factorial regression analysis of factors affecting wear scar width in basalt, carbon, and hybrid laminated composites.

Source	DF	Seq SS	Contribution	Adj SS	Adj MS	F-Value	*p*-Value
Model	32	26,885,360	99.95%	26,885,360	840,167	2098.31	0
Linear	7	23,028,035	85.61%	23,028,035	3,289,719	8216.06	0
Material	3	18,845,073	70.06%	18,845,073	6,281,691	15,688.49	0
Time (day)	3	4,139,072	15.39%	4,139,072	1,379,691	3445.77	0
Frequency (Hz)	1	43,890	0.16%	43,890	43,890	109.62	0
2-Way Interactions	15	3,469,780	12.90%	3,469,780	231,319	577.72	0
Material × Time (day)	9	3,171,705	11.79%	3,171,705	352,412	880.15	0
Material × Frequency (Hz)	3	135,310	0.50%	135,310	45,103	112.65	0
Time (day) × Frequency (Hz)	3	162,765	0.61%	162,765	54,255	135.5	0
3-Way Interactions	9	387,430	1.44%	387,430	43,048	107.51	0
Material × Time (day) × Frequency (Hz)	9	387,430	1.44%	387,430	43,048	107.51	0
Error	31	12,412	0.05%	12,412	400		
Total	63	26,897,772	100.00%				

**Table 3:** General factorial regression analysis of the factors affecting the wear scar depth in basalt, carbon, and hybrid laminated composites.

Source	DF	Seq SS	Contribution	Adj SS	Adj MS	F-Value	*p*-Value
Model	32	848,460	99.99%	848,460	26,514	7350.87	0
Linear	7	663,586	78.20%	663,586	94,798	26,281.9	0
Material	3	547,634	64.54%	547,634	182,545	50,608.9	0
Time (day)	3	108,511	12.79%	108,511	36,170	10,027.9	0
Frequency (Hz)	1	7441	0.88%	7441	7441	2063.01	0
2-Way Interactions	15	159,953	18.85%	159,953	10,664	2956.37	0
Material × Time (day)	9	137,800	16.24%	137,800	15,311	4244.86	0
Material × Frequency (Hz)	3	8034	0.95%	8034	2678	742.48	0
Time (day) × Frequency (Hz)	3	14,119	1.66%	14,119	4706	1304.8	0
3-Way Interactions	9	24,919	2.94%	24,919	2769	767.61	0
Material × Time (day) × Frequency (Hz)	9	24,919	2.94%	24,919	2769	767.61	0
Error	31	112	0.01%	112	4		
Total	63	848,572	100.00%				

**Table 4:** General factorial regression results showing the effects of material, time, and frequency on the friction coefficient.

Source	DF	Seq SS	Contribution	Adj SS	Adj MS	F-Value	*p*-Value
Model	32	3.1918	99.98%	3.19184	0.09975	5377.56	0
Linear	7	2.3632	74.02%	2.36318	0.3376	18,200.9	0
Material	3	2.1645	67.80%	2.16453	0.72151	38,898.8	0
Time (day)	3	0.0958	3.00%	0.09578	0.03193	1721.18	0
Frequency (Hz)	1	0.1029	3.22%	0.10288	0.10288	5546.6	0
2-Way Interactions	15	0.6525	20.44%	0.65249	0.0435	2345.17	0
Material Time (day)	9	0.4636	14.52%	0.46364	0.05152	2777.38	0
Material × Frequency (Hz)	3	0.0642	2.01%	0.06421	0.0214	1153.89	0
Time (day) × Frequency (Hz)	3	0.1246	3.90%	0.12463	0.04155	2239.8	0
3-Way Interactions	9	0.1762	5.52%	0.17617	0.01958	1055.34	0
Material × Time (day) × Frequency (Hz)	9	0.1762	5.52%	0.17617	0.01958	1055.34	0
Error	31	0.0006	0.02%	0.00058	1.9 × 10^−5^		
Total	63	3.1924	100.00%				

## Data Availability

The datasets presented in this article are not readily available because the data are part of an ongoing study. Requests to access the datasets should be directed to Corresponding Author.

## Abbreviations

The following abbreviations are used in this manuscript:

ANOVA	Analysis of variance
SEM	Scanning electron microscope
COF	Coefficient of friction

## References

[1] Agarwal G., Patnaik A., Sharma R.K., Agarwal J. (2014). Effect of stacking sequence on physical, mechanical and tribological properties of glass-carbon hybrid composites. Friction.

[2] Ekşi S., Danyildiz F.E., Özsoy N., Özsoy M. (2023). Tensile, bending, and impact properties of laminated carbon/aramid/glass hybrid fiber composites. Mater. Test..

[3] Karacor B., Özcanlı M. (2022). Characterization of jute/aramid hybrid composite materials using different resins. Sak. Univ. J. Sci..

[4] Özsoy M.İ., Fidan S., Bora M.Ö., Ürgün S. (2025). Understanding the damage mechanisms of basalt/carbon fiber hybrid composites under quasi-static and dynamic loadings. Polymers.

[5] Fidan S., Özsoy M.İ., Bora M.Ö., Ürgün S. (2024). Advanced hybrid composites: A comparative study of glass and basalt fiber reinforcements in erosive environments. Polym. Compos..

[6] Mayana P., Raviprakash A.V., Ali S.M., Mohammed R. (2023). Erosion wear behavior of polymer-based hybrid composites: A review. Mater. Today Proc..

[7] Dalbehera S., Acharya S.K. (2015). Impact of stacking sequence on tribological wear performance of woven jute-glass hybrid epoxy composites. Tribol. Mater. Surf. Interfaces.

[8] Rajak D.K., Wagh P.H., Linul E. (2021). Manufacturing technologies of carbon/glass fiber-reinforced polymer composites and their properties: A review. Polymers.

[9] Dash S., Satpathy M.P., Routara B.C., Pati P.R., Gantayat S. (2024). Enhancing mechanical and tribological performance of hybrid composites: An experimental study utilizing response surface methodology and firefly algorithm. Polym. Compos..

[10] Dhand V., Mittal G., Rhee K.Y., Park S.-J., Hui D. (2015). A short review on basalt fiber reinforced polymer composites. Compos. Part B Eng..

[11] Fareez U.N.M., Loudiy A., Erkartal M., Yilmaz C. (2025). Basalt fiber reinforced polymers: A recent approach to electromagnetic interference (EMI) shielding. J. Polym. Sci..

[12] Asadi A., Baaij F., Mainka H., Rademacher M., Thompson J., Kalaitzidou K. (2017). Basalt fibers as a sustainable and cost-effective alternative to glass fibers in sheet molding compound (SMC). Compos. Part B Eng..

[13] Sun H., Xiang D., Liu Z., Liu L., Harkin-Jones E., Wang M., Tan W., Luo P., Zhang J., Wang B. (2024). Failure analysis of glass fiber and basalt fiber reinforced polymer composites under an extreme environment with high-temperature, high-pressure and H2S/CO2 exposure. Polym. Compos..

[14] Saleh Mousavi-Bafrouyi S., Eslami-Farsani R., Geranmayeh A. (2020). Effect of stacking sequence on the mechanical properties of pseudo-ductile thin-ply unidirectional carbon-basalt fibers/epoxy composites. J. Ind. Text..

[15] Sahin Y., De Baets P. (2020). Effect of stacking sequence of basalt/carbon fabrics on mechanical and wear property of epoxy composites. J. Balkan Tribol. Assoc..

[16] Darshan S.M., Suresha B., Jamadar I.M. (2022). Optimization of abrasive wear parameters of halloysite nanotube reinforced silk/basalt hybrid epoxy composites using Taguchi approach. Tribol. Ind..

[17] (2004). Standard Test Method for Measuring Abrasion Using the Dry Sand/Rubber Wheel Apparatus.

[18] Kim M.T., Rhee K.Y., Lee B.H., Kim C.J. (2013). Effect of carbon nanotube addition on the wear behavior of basalt/epoxy woven composites. J. Nanosci. Nanotechnol..

[19] Chairman C.A., Kumaresh Babu S.P. (2013). Mechanical and abrasive wear behavior of glass and basalt fabric-reinforced epoxy composites. J. Appl. Polym. Sci..

[20] Pastewka L., Vakis A.I., Aghababaei R., Almqvist A., Carbone G., Chandross M., Dini D., Eder S.J., Ehrich H.J., Ewen J.P. (2025). Modeling in tribology: Recent advances, applications, and open questions. Tribol. Int..

[21] Belotti L.P., Vadivel H.S., Emami N. (2019). Tribological performance of hygrothermally aged UHMWPE hybrid composites. Tribol. Int..

[22] Zhao X., Li W., Ouyang Y., Xu W., Liu Y. (2023). Hygrothermal aging behavior and mechanical degradation of ramie/carbon fiber reinforced thermoplastic polymer hybrid composites. Ind. Crops Prod..

[23] Özsoy M.İ., Bora M.Ö., Ürgün S., Fidan S., Güleç E. (2025). Tribological performance of glass/kevlar hybrid epoxy composites: Effects of pressurized water-immersion aging under reciprocating sliding wear. Polymers.

[24] Yekani Fard M., Raji B., Pankretz H. (2020). Correlation of nanoscale interface debonding and multimode fracture in polymer composites with long-term hygrothermal effects. Mech. Mater..

[25] Guermazi N., Haddar N., Elleuch K., Ayedi H.F. (2014). Investigations on the fabrication and the characterization of glass/epoxy, carbon/epoxy and hybrid composites used in the reinforcement and the repair of aeronautic structures. Mater. Des..

[26] Guermazi N., Tarjem A.B., Ksouri I., Ayedi H.F. (2016). On the durability of FRP composites for aircraft structures in hygrothermal conditioning. Compos. Part B Eng..

[27] Sukur E.F., Elimsa S., Eskizeybek V., Avci A. (2024). Damage tolerance of basalt-reinforced multiscale composites: Effect of nanoparticle morphology and hygrothermal aging. Compos. Part B Eng..

[28] Li C., Zhang L., Wang H., Song Y., Wang J. (2024). Study of Hygrothermal Aging for Basalt Fiber/Epoxy Resin Composites Modified with CeCl3. Polymers.

[29] Bonsu A.O., Mensah C., Liang W., Yang B., Ma Y. (2022). Mechanical Degradation and Failure Analysis of Different Glass/Basalt Hybrid Composite Configuration in Simulated Marine Condition. Polymers.

[30] Kumar S., Bhowmik S. (2025). Accelerated Weathering with Humidity Effects on Physical, Surface Interfacial and Tribology Behavior of Kenaf-Pineapple Laminated Biocomposite Under Different Loading Constraint. Fibers Polym..

[31] Yorseng K., Rangappa S.M., Pulikkalparambil H., Siengchin S., Parameswaranpillai J. (2020). Accelerated weathering studies of kenaf/sisal fiber fabric reinforced fully biobased hybrid bioepoxy composites for semi-structural applications: Morphology, thermo-mechanical, water absorption behavior and surface hydrophobicity. Constr. Build. Mater..

[32] Zhang Y., Yan S., Wang X., Guan Y., Du C., Fan T., Li H., Zhai J. (2024). An experimental investigation of the mechanism of hygrothermal aging and low-velocity impact performance of resin matrix composites. Polymers.

[33] Prolongo S.G., Gude M.R., Ureña A. (2012). Water uptake of epoxy composites reinforced with carbon nanofillers. Compos. Part A Appl. Sci. Manuf..

[34] Starkova O., Buschhorn S.T., Mannov E., Schulte K., Aniskevich A. (2013). Water transport in epoxy/MWCNT composites. Eur. Polym. J..

[35] Ghabezi P., Harrison N.M. (2022). Hygrothermal deterioration in carbon/epoxy and glass/epoxy composite laminates aged in marine-based environment (degradation mechanism, mechanical and physicochemical properties). J. Mater. Sci..

[36] Russel E., Nagappan B., Karsh P., Madhu S., Devarajan Y., Suresh G., Vezhavendhan R. (2024). Effect of hygrothermal aging on novel hybrid composites: Transforming textile waste into a valuable product. Polym. Compos..

[37] Zulueta K., Burgoa A., Martínez I. (2021). Effects of hygrothermal aging on the thermomechanical properties of a carbon fiber reinforced epoxy sheet molding compound: An experimental research. J. Appl. Polym. Sci..

[38] Lu J., Zheng C., Wang L., Dai Y., Wang Z., Song Z. (2025). T700 carbon fiber/epoxy resin composite material hygrothermal aging model. Materials.

[39] (2021). Geometrical Product Specifications (GPS)—Surface Texture: Areal. Part 2: Terms, Definitions and Surface Texture Parameters.

[40] (2021). Standard Test Method for Transition Temperatures and Enthalpies of Fusion and Crystallization of Polymers by Differential Scanning Calorimetry.

[41] Tian J.W., Qi X., Guo R., Xian G. (2025). Effect of hygrothermal aging on the mechanical and frictional wear properties of carbon fiber reinforced composites. Acta Mater. Compos. Sin..

[42] Walczak M., Szala M., Pieniak D. (2022). Effect of water absorption on tribological properties of thermoplastics matrix composites reinforced with glass fibres. Adv. Sci. Technol. Res. J..

[43] Dhieb H., Buijnsters J.G., Elleuch K., Celis J.P. (2016). Effect of relative humidity and full immersion in water on friction, wear and debonding of unidirectional carbon fiber reinforced epoxy under reciprocating sliding. Compos. Part B Eng..

[44] Talib A.A.A., Jumahat A., Jawaid M., Sapiai N., Leao A.L. (2021). Effect of wear conditions, parameters and sliding motions on tribological characteristics of basalt and glass fibre reinforced epoxy composites. Materials.

[45] Sang L., Wang Y., Wang C., Peng X., Hou W., Tong L. (2019). Moisture diffusion and damage characteristics of carbon fabric reinforced polyamide 6 laminates under hydrothermal aging. Compos. Part A Appl. Sci. Manuf..

[46] Wong K.J., Johar M., Koloor S.S.R., Petrů M., Tamin M.N. (2020). Moisture absorption effects on Mode II delamination of CF/epoxy composites. Polymers.

[47] Song S., Zhu Z., Du S., Li Y., Liu C. (2024). Research on Polymer Wear under Water Conditions: A Review. Lubricants.

[48] Huang Y., Cai M., He C., Si C., Li L., Fan X., Zhu M. (2023). Basalt fiber as a skeleton to enhance the multi-conditional tribological properties of epoxy coating. Tribol. Int..

[49] Oliveira M.S., da Luz F.S., Pereira A.C., Costa U.O., Bezerra W.B.A., da Cunha J.d.S.C., Lopera H.A.C., Monteiro S.N. (2022). Water Immersion Aging of Epoxy Resin and Fique Fabric Composites: Dynamic–Mechanical and Morphological Analysis. Polymers.

[50] Rocha I.B.C.M., Raijmaekers S., Nijssen R.P.L., van der Meer F.P., Sluys L.J. (2017). Hygrothermal ageing behaviour of a glass/epoxy composite used in wind turbine blades. Compos. Struct..

[51] Choi S., Douglas E.P. (2010). Complex hygrothermal effects on glass transition of an epoxy-amine thermoset. ACS Appl. Mater. Interfaces.

[52] Mu W., Li S., Zhang S., Chen X., Sun Y., Zhou K., Qin G. (2024). Effect of hygrothermal aging on mechanical properties of continuous flax fiber composites. Polym. Compos..

[53] Santos da Silva G.A., Moraes d’Almeida J.R., Taissum Cardoso D.C. (2024). Investigation on moisture absorption behavior on GFRP and neat epoxy systems in hygrothermal salt fog aging. Compos. Part B Eng..

